# Synthesis of Novel Selenocyanates and Evaluation of Their Effect in Cultured Mouse Neurons Submitted to Oxidative Stress

**DOI:** 10.1155/2020/5417024

**Published:** 2020-05-28

**Authors:** Tiago E. A. Frizon, José H. Cararo, Sumbal Saba, Gustavo C. Dal-Pont, Monique Michels, Hugo C. Braga, Tairine Pimentel, Felipe Dal-Pizzol, Samira S. Valvassori, Jamal Rafique

**Affiliations:** ^1^Department of Energy and Sustainability, Federal University of Santa Catarina (UFSC), Araranguá, 88906-072 SC, Brazil; ^2^Translational Psychiatry Laboratory, Graduate Program in Health Sciences, University of Southern Santa Catarina (UNESC), Criciúma, 88806-000 SC, Brazil; ^3^Center for Natural and Human Sciences-CCNH, Federal University of ABC (UFABC), Santo André, 09210-580 SP, Brazil; ^4^Laboratory of Experimental Pathophysiology, Graduate Program in Health Sciences, Health Sciences Unit, University of Southern Santa Catarina (UNESC), Criciúma. 88806-000 SC, Brazil; ^5^Federal University of São Paulo (UNIFESP), São José dos Campos, 12231-280 SP, Brazil; ^6^Institute of Chemistry, Federal University of Mato Grosso do Sul (UFMS), Campo Grande, 79074-460 MS, Brazil

## Abstract

Herein, we report the synthesis of novel selenocyanates and assessment of their effect on the oxidative challenge elicited by hydrogen peroxide (H_2_O_2_) in cultured mouse neurons. First, *α*-methylene-*β*-hydroxy esters were prepared as precursors of allylic bromides. A reaction involving the generated bromides and sodium selenocyanate was conducted to produce the desired selenocyanates (3a-f). We next prepared cultures of neurons from 7-day-old mice (*n* = 36). H_2_O_2_ (10^−5^ M) was added into the culture flasks as an oxidative stress inducer, alone or combined with one of each designed compounds. (PhSe)_2_ was used as a positive control. It was carried out assessment of lipid (thiobarbituric acid reactive species, 4-hydroxy-2′-nonenal, 8-isoprostane), DNA (8-hydroxy-2′-deoxyguanosine), and protein (carbonyl) modification parameters. Finally, catalase and superoxide dismutase activities were also evaluated. Among the compounds, 3b, 3d, and 3f exhibited the most pronounced pattern of antioxidant activity, similar to (PhSe)_2_. These novel aromatic selenocyanates could be promising to be tried in most sophisticated *in vitro* studies or even at the preclinical level.

## 1. Introduction

Selenium (Se) is a trace element regarded essential for humans and other mammalians [1]. The main biological role of this metalloid is related to its incorporation into selenoproteins, most of which participate in redox homeostasis (particularly glutathione peroxidase [GPx, EC 1.11.1.9] and thioredoxin reductase [EC 1.8.1.9]), metabolism of thyroid hormones, biosynthesis of other Se-containing proteins [2], and in the spermatogenesis [3]. In addition, adequate supplementation with Se contributes to the detoxification of heavy metals, including mercury and related compounds [4]. At least in part, this effect is ascribable to the marked antioxidant property of Se and its organic analogues [5].

**Figure 1:**
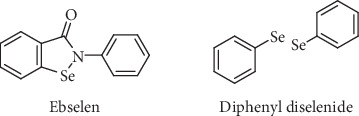
Structure of Ebselen and diphenyl diselenide.

In this regard, a number of papers have pointed out the synthetic versatility of compounds termed organochalcogens [6–13]. Selenium-containing organochalcogenides seem to be of great therapeutic relevance, mostly due to the ability of these compounds to mimic natural substances with antioxidant, antitumor, antimicrobial, and antiviral activities [14–20]. One of the most known drugs of such class is called Ebselen (Figure 1), which presents GPx-like activity and has been searched for therapy of several human disorders [21]. In relation with the current pandemic of coronavirus (COVID-19), a very interesting study just appeared in the literature where the organoselenium compound, Ebselen, presented the strongest antiviral effect at a concentration of 10 *μ*M treatment in COVID-19 virus-infected Vero cells [22]. Diphenyl diselenide ((PhSe)_2_) is another organoselenium compound with GPx-like activity (Figure 1) [23].

By considering the biological roles of these compounds, the development of new and efficient routes for the synthesis of organoselenides is a tempting research area [12, 24, 25]. Therefore, as an additional step of the ongoing efforts aiming at the design of novel organoselenides and medicinal chemistry [26–28], the present study reports the synthesis of novel aromatic selenocyanates, comprising a second generation of chalcogenide esters. Furthermore, assessment of the effect of these compounds on the oxidative challenge elicited by hydrogen peroxide (H_2_O_2_) in mixed cultures of mouse neurons was undertaken, in order to provide insightful cues on their biological activity.

## 2. Materials and Methods

### 2.1. General Procedure for the Synthesis of Allylic Bromide 2

5.0 mmol of LiBr was added to a stirred solution of 2.5 mmol of *α*-methylene-*β*-hydroxy esters (Morita-Baylis-Hillman adduct) 1 in 10.0 mL of acetonitrile at 0-5°C, followed by the addition of 6.3 mmol of 96% H_2_SO_4_. Subsequently, the reaction mixture was allowed to attend the room temperature and the stirring was continued until the complete consumption of 1 (monitored my by TLC). The reaction mixture was diluted by CH_2_Cl_2_ (20 mL), and the organic phase was successively extracted with H_2_O, saturated NaHCO_3_, brined, and dried over MgSO_4_. The organic phase was concentrated under reduced pressure, and the resulting residue was purified by column chromatography (hexane/ethyl acetate 9 : 1) using flash silica gel, resulting in the corresponding allylic bromides 2a-f. (Figures S13–S18, FTIR for compound 2a-f in Supplementary Materials, pg 11–13).

### 2.2. General Procedure for the Synthesis of Allylic Selenocyanates 3

To a stirred solution of allylic bromide 2a-f (1.0 mmol) in 4.0 mL of acetone/H_2_O (4 : 1 *v*/*v*) at 25°C was added 1.25 mmol of KSeCN. After stirring for 10 h, the final mixture was diluted with CH_2_Cl_2_ and washed with H_2_O and brine. The organic extract was dried over Na_2_SO_4_, filtered, and concentrated under reduced pressure. The resulting residue was purified by chromatography (hexane/ethyl acetate 9 : 1) to give the corresponding compounds 3a-f.

### 2.3. Methyl (*Z*)-3-phenyl-2-(selenocyanatomethyl)acrylate (3a)

Yield 96%; m.p. 72-73°C. IR (KBr): *ν*max/cm^−1^ 305, 3003, 2956, 2935, 2849, 2141, 1693, 1619, 1435, 1346, 1255, 1077, 754, 482. 1H NMR (200 MHz, CDCl_3_): *δ* 7.88 (s, 1H), 7.59–7.36 (m, 5H), 4.17 (s, 2H), 3.86 (s, 3H). 13C NMR (50 MHz, CDCl_3_): *δ* 166.8, 143.0, 133.7, 129.5, 129.1, 128.8, 126.9, 102.4, 52.5, 25.5. Anal. Calcd for C_12_H_11_NO_2_Se: C, 51.44; H, 3.96; Br, 22.25; N, 5.00; O, 11.42; Se, 28.18. Found: C, 50.53; H, 3.70. (Figure S1, 1H and 13C NMR Spectra and Figure S7, FTIR for compound 3a in Supplementary Materials, pg 2 and 8, respectively).

### 2.4. Methyl (*Z*)-3-(4-bromophenyl)-2-(selenocyanatomethyl)acrylate (3b)

Yield 86%; m.p. 72-74°C: *ν*max/cm^−1^ 3062, 2952, 2840, 2156, 1710, 1625, 1487, 1435, 1273, 1198, 1154, 1068, 1008, 808, 503. 1H NMR (400 MHz, CDCl_3_): *δ* 7.82 (s, 1H), 7.61 (d, *J* =8.4 Hz, 2H), 7.31 (d, *J* = 8.0 Hz, 2H), 4.14 (s, 2H), 3.89 (s, 3H). 13C NMR (101 MHz, CDCl_3_): *δ* 166.8, 141.9, 132.7, 132.4, 130.8, 127.8, 124.2, 102.4, 52.9, 25.4. Anal. Calcd for C_12_H_10_BrNO_2_Se: C, 40.14; H, 2.81; Br, 22.25; N, 3.90; O, 8.91; Se, 21.99. Found: C, 40.13; H, 2.80. (Figure S2, 1H and 13C NMR Spectra and Figure S8, FTIR for compound 3b in Supplementary Materials, pg 3 and 8, respectively).

### 2.5. Methyl (*Z*)-3-(2-bromophenyl)-2-(selenocyanatomethyl)acrylate (3c)

Yield 98%; white solid, m.p. 71.5-73.0°C. IR (KBr): *ν*max/cm^−1^ 3032, 2953, 2926, 2850, 2150, 1678, 1438, 1363, 1295, 1090, 758, 586. 1H NMR (400 MHz, CDCl_3_): *δ* 7.88 (s, 1H), 7.66 (dd, *J* = 8.1, 1.3 Hz, 1H), 7.48–7.39 (m, 2H), 7.31–7.26 (m, 1H), 4.00 (s, 2H), 3.91 (s, 3H). MHz, CDCl_3_): *δ* 166.7, 142.1, 134.5, 133.2, 130.9, 130.0, 129.1, 127.8, 123.9, 102.7, 52.9, 25.5. Anal. Calcd for C_12_H_10_BrNO_2_Se: C, 40.14; H, 2.81; Br, 22.25; N, 3.90; O, 8.91; Se, 21.99. Found: C, 40.12; H, 2.78. (Figure S3, 1H and 13C NMR Spectra and Figure S9, FTIR for compound 3c in Supplementary Materials, pg 4 and 9, respectively).

### 2.6. Methyl (*Z*)-3-(4-chlorophenyl)-2-(selenocyanatomethyl)acrylate (3d)

Yield 94%; white solid, m.p. 73-75°C. IR (KBr): *ν*max/cm^−1^ 3088, 2952, 2854, 2149, 1716, 1583, 1453, 1287, 1197, 1082, 793. 1H NMR (400 MHz, CDCl_3_): *δ* 7.84 (s, 1H), 7.45 (d, *J* = 8.7 Hz, 2H), 7.37 (d, *J* = 8.2 Hz, 2H), 4.15 (s, 2H), 3.88 (s, 3H). 13C NMR (101 MHz, CDCl_3_): *δ* 166.8, 141.8, 135.8, 132.2, 130.6, 129.3, 127.7, 102.4, 52.8, 25.4. Anal. Calcd for C_12_H_10_ClNO_2_Se: C, 45.81; H, 3.20; Cl, 11.27; N, 4.45; O, 10.17; Se, 25.10. Found: C, 45.80; H, 3.18. (Figure S4, 1H and 13C NMR Spectra and Figure S10, FTIR for compound 3d in Supplementary Materials, pg 5 and 9, respectively).

### 2.7. Methyl (*Z*)-3-(2,4-dichlorophenyl)-2-(selenocyanatomethyl)acrylate (3e)

Yield 89%; white solid, m.p. 75.0-78.0°C. IR (KBr): *ν*max/cm^−1^ 3088, 2952, 2854, 2149, 1716, 1583, 1435, 1287, 1167, 1082, 763. 1H NMR (400 MHz, CDCl_3_): *δ* 7.86 (s, 1H), 7.50 (d, *J* = 0.9 Hz, 1H), 7.41–7.37 (m, 2H), 3.98 (s, 2H), 3.91 (s, 3H). 13C NMR (101 MHz, CDCl_3_): *δ* 166.5, 138.8, 136.3, 135.0, 131.1, 130.8, 130.1, 130.0, 127.7, 102.6, 53.3, 25.4. Anal. Calcd for C_12_H_9_Cl_2_NO_2_Se: C, 41.29; H, 2.60; Cl, 20.31; N, 4.01; O, 9.17; Se, 22.62. Found: C, 41.31; H, 2.58. (Figure S5, 1H and 13C NMR Spectra and Figure S11, FTIR for compound 3e in Supplementary Materials, pg 6 and 10, respectively).

### 2.8. Methyl (*Z*)-3-(4-nitrophenyl)-2-(selenocyanatomethyl)acrylate (3f)

Yield 95%; yellow solid, m.p. 83-84°C. IR (KBr): *ν*max/cm^−1^ 3106, 2955, 2846, 2150, 1721, 1599, 1516, 1429, 1342, 1272, 1202, 1158, 854, 769. 1H NMR (400 MHz, CDCl_3_): *δ* 8.32 (d, *J* = 8.9 Hz, 2H), 7.93 (s, 1H), 7.61 (d, *J* = 8.8 Hz, 2H), 4.10 (s, 2H), 3.92 (s, 3H). 13C NMR (101 MHz, CDCl_3_): *δ* 166.2, 147.9, 140.3, 140.1, 130.5, 130.0, 124.1, 102.1, 53.0, 24.8. Anal. Calcd for C_12_H_10_N_2_O_4_Se: C, 44.32; H, 3.10; N, 8.61; O, 19.68; Se, 24.28. Found: C, 44.29; H, 3.11. (Figure S6, 1H and 13C NMR Spectra and Figure S12, FTIR for compound 3f in Supplementary Materials, pg 7 and 10, respectively).

### 2.9. Animals

Thirty-six, 7-day-old mice (*Mus musculus*; Balb/C strain) were obtained from the Central Animal House of the University of Southern Santa Catarina (UNESC). Animals received *ad libitum* water and chow and were kept in a colony room with 21 ± 1°C temperature and a 12 hours light/dark cycle. Experimental groups were as follows: positive control (culture medium and sample); negative control or stress (culture medium, sample, and H_2_O_2_); and a group for each tested 3a–f compounds (culture medium, sample, H_2_O_2_, and the corresponding compound—seven groups); it was used four animals per group. Mice were killed by decapitation without anesthesia, the skull was opened, and the total brain was excised and cleaned. All experimental procedures were performed with approval by the UNESC's Ethical Committee (protocol # 012/2016-1).

### 2.10. Cell Culture and Hydrogen Peroxide Challenge

Immediately after euthanasia of the animals, the brain was placed in a chamber with constant air flux and ultraviolet illumination for incubation. Cells from both cerebral hemispheres were dissociated in phosphate-buffered saline (0.9%) and plated at a density of 10^5^ cells/cm^2^ in 75 cm^2^ culture flasks with Dulbecco's Modified Eagle's Medium supplemented with 10% fetal bovine serum and 20% antibiotics. H_2_O_2_ (10^−5^ M) was added soon after as an oxidative stress inducer according to the reference [29]. Each 3a–f compound was also added to the medium, aiming to reach a 10 *μ*M concentration, based on the study carried out by Posser and coworkers [23]. (PhSe)_2_, which presents GPx-like activity, was used as a positive control [23]. The plate was kept in a carbon dioxide (CO_2_)-incubator during 24 hours. Thereafter, samples were stored at –80°C for the subsequent analyses.

### 2.11. Assessment of Lipid Peroxidation

Quantification of thiobarbituric acid reactive species (TBARS) was carried out on the basis of malondialdehyde (MDA) content through the reference [30]. Briefly, samples (200 *μ*L aliquot) were mixed with 1 mL 10% trichloroacetic acid and 1 mL 0.67% thiobarbituric acid and heated in boiling water during 30 minutes. MDA equivalents absorbance was measured at *λ* = 532 nm, using 1,1,3,3-tetramethoxypropane as standard. Data were expressed as MDA equivalents (nmol/mg protein).

4-hydroxy-2-nonenal (4-HNE) content was determined using the assay kit from Cell Biolabs (Cell Biolabs, Inc., San Diego, California, USA). 8-isoprostane (8-ISO) level was measured using the ACE™ Competitive EIAs Kit (Cayman) with 8-isoprostane-acetylcholinesterase (EC 3.1.1.7) conjugate as a tracer and 8-isoprostane-specific rabbit antiserum. Adducts of 4-HNE with lysine, histidine, or cysteine residues in proteins were quantified according to the immunoassay described by Kimura and coworkers [31].

### 2.12. DNA and Protein Modification Parameters

Nuclear DNA was isolated from the cells using the PureGenome™ On-Spot Tissue DNA Kit (EMD Millipore, Burlington, Massachusetts, USA). DNA content in the extracts was measured by using NanoDrop (Thermo Fisher Scientific, Waltham, Massachusetts, USA). Levels of 8-hydroxy-2′-deoxyguanosine (8-OHdG), a compound generated by oxidation of 2′-deoxyguanosine residues, were determined using the OxiSelect™ Oxidative DNA Damage ELISA Kit (Cell Biolabs, Inc., San Diego, USA).

Protein oxidative damage was estimated on the basis of carbonyl content determination according to the method described by Levine and coworkers [32]. Protein precipitation was conducted by the addition of 20% trichloroacetic acid to the samples (400 *μ*L aliquot), which were then dissolved in a diphenylhydrazine (DNPH) solution. Data were expressed as nmol/mg protein. Sample absorbance was read at *λ* = 370 nm.

### 2.13. Catalase Activity

Catalase (CAT; EC 1.11.1.6) activity was assessed on the basis of *in vitro* H_2_O_2_ decomposition according to the standardized method [33]. Brain tissue was sonicated in 50 mM phosphate buffer (pH 7.0), and the resulting suspension was submitted to centrifugation (3,000 g for 10 minutes). A 20 *μ*L sample aliquot was added to 980 *μ*L substrate mixture, which contained 0.3 mL H_2_O_2_ in 50 mL 0.05 M phosphate buffer (pH 7.0). Initial and final absorbance values were recorded at *λ* = 240 nm after 1 and 6 minutes, respectively. A standard curve was established using purified CAT (Sigma-Aldrich, St. Louis, Missouri, United States) at the same experimental conditions of the samples.

### 2.14. Superoxide Dismutase Activity

Measurement of superoxide dismutase (SOD; EC 1.15.1.1) activity was performed based on its ability to spontaneously inhibit oxidation of adrenaline to adrenochrome [34]. Sodium carbonate buffer (2.78 mL; 0.05 mM; pH 10.2), 100 *μ*L EDTA (1.0 mM), and 20 *μ*L supernatant or sucrose solution (blank) were incubated at 30°C for 45 minutes. Thereafter, the reaction was initiated by adding 100 *μ*L adrenaline solution (9.0 mM). The change in absorbance was recorded at *λ* = 480 nm for 8 minutes. Temperature was maintained at 30°C throughout the assay procedure. One unit of SOD produced 50% of adrenaline auto-oxidation. Data were expressed as units/mg protein.

### 2.15. Determination of Protein Content in the Samples

Biochemical analyses were related to the protein content in the samples, for normalization. A 10 *μ*L aliquot of each sample was used in this procedure. Measurements were carried out according to the Peterson's method [35]. Bovine serum albumin was used as standard.

### 2.16. Statistical Analysis

Data were expressed as mean ± standard deviation (S.D.). Differences between groups were analyzed by two-way analysis of variance (ANOVA) followed by Tukey's *post hoc* test. The software used for the comparisons was the Statistical Package for the Social Sciences (SPSS) 20 (IBM, Armonk, New York, USA). Differences were rated as statistically significant at *p* < 0.05.

## 3. Results

### 3.1. Synthesis of the Selenocyanates (3)

*α*-Methylene-*β*-hydroxy esters for organic synthesis were readily prepared by the Morita-Baylis-Hillman reaction. Such compounds are building blocks for the synthesis of several substances, by converting their hydroxyl group into acetates, bromides, and thiocyanates, acting like acceptors in many useful synthetic approaches. Thus, allylic bromides (2) were obtained according to the method described by Ferreira and coworkers [36]. In this procedure, lithium bromide (LiBr) and H_2_SO_4_ were added to a previously stirred solution of Baylis-Hillman adducts (i.e., *α*-methylene-*β*-hydroxy esters, 1) in acetonitrile at 0-5°C (Table 1). The reaction mixture stirred until the consumption of the starting material. After the completion of the reaction, dichloromethane (CH_2_Cl_2_) was added and washed with water, saturated with sodium bicarbonate (NaHCO_3_) and brine, dried over magnesium sulfate (MgSO_4_), filtered, and concentrated under reduced pressure. The resulting residue was purified by column chromatography to yield the corresponding 2-bromomethyl-2-alkenoates 2.

In the next step, a reaction was conducted involving the generated 2 and potassium selenocyanate (KSeCN) to produce the desired allylic selenocyanates [37]. Briefly, the process occurs by nucleophilic displacement of bromide with KSeCN in acetone, without the use of an external base. More specifically, aromatic-substituted allylic selenocyanates (3) could be obtained by mixing the corresponding bromides (2) with 1.25 molar equiv. of KSeCN in acetone at 25°C in 10 min. The corresponding products (3) were then isolated in high yields after purification in a short plug of silica gel using acetone as eluent (Table 1). Assignment of the organoselenium structures (3a–f) was based on the characteristic signals for the selenocyanate (SeCN) functional group at infrared (IR; sharp band at 2140–2157 cm^−1^) and carbon-13 nuclear magnetic resonance (^13^C NMR, 102 ppm) spectra of all purified products (Supplementary Materials).

### 3.2. Effect of Different Selenocyanates (3) towards Lipid Peroxidation Elicited by H_2_O_2_

**Figure 2:**
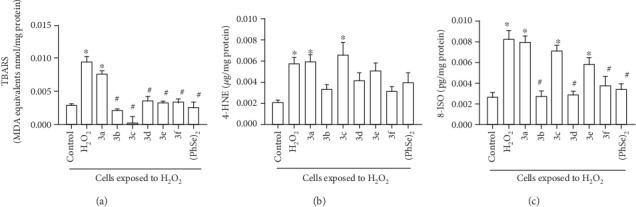
Thiobarbituric acid reactive species (TBARS, a), 4-hydroxy-2′-nonenal (4-HNE, b) and 8-isoprostane (8-ISO, c) levels in cultures of neurons obtained from 7-day-old-mice exposed to hydrogen peroxide (H_2_O_2_) alone or in the presence of organoselenium compounds (diphenyl diselenide ((PhSe)_2_) or one of each tested selenocyanates (3a–f)). The concentration of each organoselenium compound in the medium was 10 *μ*M. Data were expressed as malondialdehyde equivalents nmol per milligram protein (MDA equivalents nmol/mg protein—TBARS), micrograms per milligram protein (*μ*g/mg protein – 4-HNE) and picograms per milligram protein (pg/mg protein – 8-ISO). *n* = 4 animals per group. ^∗^*p* < 0.05, as compared to the control group; #*p* < 0.05, as compared to the stress group. (Tukey's *post hoc* test).

Initially, it was performed measurement of TBARS level in the samples (Figure 2(a)). As expected, TBARS content was significantly increased in the cell cultures only receiving H_2_O_2_ (stress group), as compared to the control group (*p* < 0.05). In addition, it was detected increased MDA levels in the cells subjected to the oxidative challenge and receiving Compound 3a as well, in comparison to the control group (*p* = 0.000026). In contrast, addition of Compound 3b, Compound 3c, Compound 3d, Compound3e, Compound 3f, or (PhSe)_2_ to the medium significantly reduced TBARS levels in cells exposed to H_2_O_2_, as compared to the cultures from stress group (*p* < 0.05).

The next step in the assessment of lipid peroxidation of the samples was the determination of the 4-HNE content (Figure 2(b)). Significant increases in this parameter were detected in cell cultures exposed to H_2_O_2_ alone or in combination with Compound 3a or Compound 3c, as compared to control cells (H_2_O_2_: *p* = 0.01002; Compound 3a: *p* = 0.0053; Compound 3c: *p* = 0.00103). However, 4-HNE levels in cells exposed to H_2_O_2_ whose medium received Compound 3b, Compound 3d, and Compound 3f or (PhSe)_2_ exhibited a trend to decrease in comparison to cells from stress group (Compound 3b: *p* = 0.22; Compound 3d: *p* = 0.74; Compound 3e: *p* = 0.99; Compound 3f: *p* = 0.16; (PhSe)_2_: *p* = 0.61).

Finally, 8-ISO content was measured to provide further insight on the lipid peroxidation elicited by H_2_O_2_ (Figure 2(c)). Significantly increased levels of such marker were detected in cells exposed to H_2_O_2_ alone or combined with Compound 3a, Compound 3c, or Compound 3e, as compared to control cells (H_2_O_2_: *p* = 0.000004; Compound 3a: *p* = 0.000008; Compound 3c: *p* = 0.00019; Compound 3e: *p* = 0.014). In contrast, addition of Compound 3b, Compound 3d, Compound 3f, or (PhSe)_2_ into the medium of cells exposed to H_2_O_2_ produced a decrease in the 8-ISO content, as compared to cells submitted to the oxidative challenge in a selenocyanate free medium (Compound 3b: *p* = 0.000004; Compound 3d: *p* = 0.000007; Compound 3f: *p* = 0.00016; (PhSe)_2_: *p* = 0.000051).

### 3.3. Effect of Different Selenocyanates on the H_2_O_2_-Induced Oxidative Damage against DNA and Proteins

**Figure 3:**
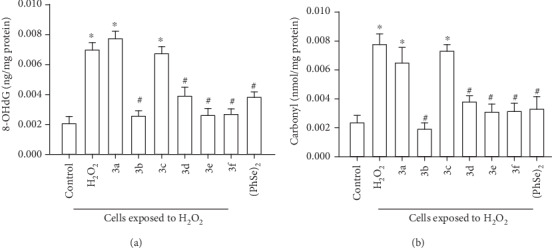
8-Hydroxy-2′-deoxyguanosine (8-OHdG, a) and protein carbonyl (b) levels in cultures of neurons obtained from 7-day-old mice exposed to hydrogen peroxide (H_2_O_2_) alone or in the presence of organoselenium compounds (diphenyl diselenide ((PhSe)_2_) or one of each tested selenocyanates (3a–f)). The concentration of each organoselenium compound in the medium was 10 *μ*M. Data were expressed as nanograms per milligram protein (ng/mg protein) and nanomoles per milligram protein (nmol/mg protein). *n* = 4 animals per group. ^∗^*p* < 0.05, as compared to the control group; #*p* < 0.05, as compared to the stress group. (Tukey's *post hoc* test).

In the present contribution, DNA damage was estimated on the basis of 2′-deoxyguanosine residue oxidation into 8-OHdG. There was an increase in the levels of this by-product in cells receiving H_2_O_2_ alone or combined with Compound 3a or Compound 3c, in comparison to control cells (H_2_O_2_: *p* < 0.05; Compound 3a: *p* < 0.05; Compound 3c: *p* < 0.05). Nevertheless, reduced 8-OHdG content was detected in the cells submitted to the oxidative challenge but receiving one of each remaining selenocyanates in their medium, as compared to cell cultures from stress group (Compound 3b: *p* < 0.05; Compound 3d: *p* = 0.00044; Compound 3e: *p* < 0.05; Compound 3f: *p* = 0.000001; (PhSe)_2_: *p* = 0.00025).  Figure 3(a) depicts data obtained with the determination of 8-OHdG levels in the cell cultures.

Additionally, increased carbonyl content—an important parameter correlated with protein damage, was found in cells receiving H_2_O_2_ alone or in combination with Compound 3a or Compound 3c, in comparison to control cells (H_2_O_2_: *p* = 0.000022; Compound 3a: *p* = 0.0018; Compound 3c: *p* = 0.00012). In contrast, the addition of Compound 3b, Compound 3d, Compound 3e, Compound 3f, or (PhSe)_2_ to the cell medium exposed to H_2_O_2_ significantly mitigated increased carbonyl levels, as compared to the cell culture submitted to oxidative challenge with no addition of selenocyanate (Compound 3b: *p* = 0.000004; Compound 3d: *p* = 0.0021; Compound 3e: *p* = 0.00027; Compound 3f: *p* = 0.00028; (PhSe)_2_: *p* = 0.00047).  Figure 3(b) shows the findings obtained with the determination of carbonyl content in the cultures.

### 3.4. Effect of the Synthesized Compounds on Activity of Antioxidant Enzymes

**Figure 4:**
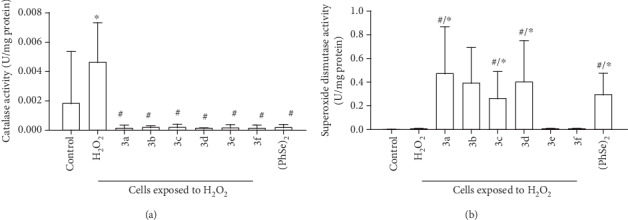
Catalase (a) and superoxide dismutase (b) activities in cultures of neurons obtained from 7-day-old mice exposed to hydrogen peroxide (H_2_O_2_) alone or in the presence of organoselenium compounds (diphenyl diselenide ((PhSe)_2_) or one of each tested selenocyanates (3a–f)). The concentration of each organoselenium compound in the medium was 10 *μ*M. Data were expressed as enzyme units per milligram protein (U/mg protein). *n* = 4 animals per group. ^∗^*p* < 0.05, as compared to the control group; #*p* < 0.05, as compared to the stress group. (Tukey's *post hoc* test).

Figure 4 depicts the effect of selenocyanates (3) on the CAT activity. Oxidative challenge elicited by H_2_O_2_ produced a significant increase in this parameter (*p* < 0.05, Figure 4(a)). In comparison to neurons only receiving H_2_O_2_, decreased CAT activity was detected in all cultures in which there was an addition of any organoselenium compound in particular (Figure 4(a); *p* < 0.05). In contrast, no significant differences in SOD activity were found between control cultures and neurons receiving H_2_O_2_ (Figure 4(b); *p* = 1.00). Interestingly, the addition of Compound 3a, Compound 3b, Compound 3c, Compound 3d, or (PhSe)_2_ elicited upregulation of SOD activity, as compared to the control or H_2_O_2_ cultures (Figure 4(b); *p* < 0.05). A similar effect was not observed when Compound 3e or Compound 3f was added into the medium (Figure 4(b); *p* > 0.05).

## 4. Discussion

Administration of compounds bearing the selenocyanate functional group in their structure was significantly associated to inhibition of lipid peroxidation and enhancement of antioxidant enzyme defences in the liver of mice receiving cadmium [38]. In addition, antioxidant activity was implicated in at least part of the therapeutic properties of organoselenium compounds [39, 40]. This effect was further showed in studies performed with cell culture [41–43] and animal species such as rats [44, 45], fishes [46], *Drosophila melanogaster* [47], and *Caenorhabditis elegans* [48].

The present study reported that cells submitted to oxidative challenge with H_2_O_2_ in culture medium containing Compound 3b, Compound 3c, Compound 3d, Compound 3e, Compound 3f, or the control compound (PhSe)_2_ exhibited lower TBARS levels than cells in similar conditions but without addition of selenocyanates; Compound 3a was the only that failed to prevent this oxidative modification elicited by H_2_O_2_. Furthermore, all compounds failed to prevent an increase in the 4-HNE levels elicited by H_2_O_2_. However, a trend to decrease in such parameter was observed in cells subjected to oxidative challenge in the presence of Compound 3b, Compound 3d, Compound 3f, or (PhSe)_2_. With respect to 8-ISO content, Compound 3a, Compound 3c, or Compound 3e was not effective in normalization of the aberrant marker levels detected in cells into a pro-oxidant culture environment. Presence of Compound 3b, Compound 3d, Compound 3f, or (PhSe)_2_ in the medium collaborated to lowering 8-ISO levels in cells at oxidative challenge. In addition, increased 8-OHdG levels were detected in cells exposed to H_2_O_2_ alone or combined with Compound 3a or Compound 3c, whereas decreased content of this marker was found in cells receiving Compound 3b, Compound 3d, Compound 3e, Compound 3f, or (PhSe)_2_. Similarly, it was showed higher carbonyl content in cells receiving H_2_O_2_ alone or combined with Compound 3a or Compound 3c; in contrast, presence of Compound 3b, Compound 3d, Compound 3e, Compound 3f, or (PhSe)_2_ in the medium triggered a significant decrease in this parameter. All the synthesized compounds were implicated in significant attenuation of CAT activity, whereas four compounds (Compounds 3a-d) and (PhSe)_2_ produced upregulation of SOD activity when added to the cultured neurons exposed to H_2_O_2_.

Thus, Compound 3b, Compound 3d, and Compound 3f exhibited the most favorable antioxidant profile, potentially with higher scavenging activity towards reactive oxygen species. Since (PhSe)_2_ presents GPx-like antioxidant activity and so was used in the present study as reference [23], the three aforementioned compounds could in part act mimicking this enzyme activity. On the other hand, Compound 3a and Compound 3c were the substances with the worst performance. Compound 3e, in turn, showed a moderate antioxidant profile, due to the fact of this compound did not counteract alterations in the levels of 4-HNE and 8-ISO. Regarding R groups attached to the aromatic ring bound to a five-carbon branch bearing a selenocyanate group, nitro or bromine functional group at *para* orientation and chlorine group at *orto* orientation could be pivotal to the antioxidant profile of the molecules designed with such R groups. Accordingly, compounds with bromine group at *orto* orientation or chlorine group at *para* orientation showed weaker antioxidant activity, which substantiates the importance of R group position on the activity of each selenocyanate. Indeed, minimal structural alterations in selenocyanates may strictly influence their antioxidant activity, as shown in the paper performed by Ibrahim and coworkers [40]. The precise mechanisms underpinning marked differences in the antioxidant profile of compounds with so similar structure are unknown, but they may be partly driven by the experimental observation indicating that bromine is a weaker functional group to withdraw electrons, as compared to chlorine or nitro groups, keeping a higher electron density at the selenocyanate group [49]. Although (PhSe)_2_ is not a selenocyanate, its antioxidant activity may be related to the phenylselenyl group, which was recently described by Vogt and colleagues [50] as pivotal for the antioxidant roles of 7-chloro-4-phenylselenyl-quinoline.

Bis(2-hydroxyphenyl) diselenide and other diphenyl diselenides possess remarkable radical trapping activity, preventing an increase in the contents of protein carbonyls and lipid hydroperoxides elicited by oxidative imbalances [51]. The reference compound, (PhSe)_2_, has been tested to manage several pathological conditions characterized by oxidative disturbances. To illustrate, the antiviral property of the compound was associated to its antioxidant action in mice infected with type 2 *Herpes simplex* virus in the paper performed by Sartori and coworkers [52]; this antioxidant activity was characterized by a decrease in MDA content and alleviation of CAT and SOD inhibition—parameters assessed in the present study. Furthermore, (PhSe)_2_ was showed to counteract reductions in the content of total thiol and activity of antioxidant enzymes as well the release of reactive oxygen species and TBARS elicited by acute intoxication with manganese in *Drosophila melanogaster* [47]. The compound was also effective to mitigate protein and lipid oxidative modifications and collaborated to normalize CAT and SOD activities in the colon of rats submitted to dextran sulfate-induced colitis [53]. Moreover, (PhSe)_2_ markedly decreased levels of reactive oxygen species and alleviated inflammation in the spleen of rodents chronically infected with *Toxoplasma gondii* [54]. Therefore, our findings on the effect of (PhSe)_2_ corroborate its previously reported antioxidant activity.

Since marked antioxidant effect was assigned to Compound 3b, Compound 3d, and Compound 3f and the fact of antioxidant activity was showed to contribute to the therapeutic properties of organoselenium compounds [40], it is expected that these novel compounds could be promising in experimental studies performed in pathological scenarios in which oxidative stress is strongly implicated. We cannot ascertain the precise mechanisms involved in this antioxidant effect. Possible ways may include the direct trapping (and eventual decomposition) of the reactive oxygen species in the cell medium, mimicking the activity of antioxidant enzymes, as described for (PhSe)_2_ and Ebselen. On the other hand, compounds can act indirectly, triggering signaling cascades aiming to abrogate oxidative stress. Indeed, several antioxidants strongly activate a signaling molecule involved in response to oxidative stress and cell survival termed nuclear factor erythroid 2-related factor 2 (Nrf2) [55]. For example, supplementation with the antioxidant *p,p*′-methoxyl-diphenyl diselenide was implicated in Nrf2 activation in frontal cortex of rats submitted to experimental pain-depression dyad [44]. 3-Selena-1-dethiacephem and (PhSe)_2_were also involved in Nrf2 pathway activation, contributing for the antioxidant roles of these organoselenium compounds [56, 57]. Thus, Nrf2 activation by the novel selenocyanates cannot be ruled out in the present study, potentially substantiating the reported antioxidant properties.

It is noteworthy that selenocyanates have been tested for the therapy of oxidative stress-driven diseases, including cancer [58]. In this regard, supplementation with 1,4-phenylenebis(methylene)selenocyanate (*p*-XSC) was showed to abrogate the tumorigenesis process triggered by nitrosamine and enhance antioxidant defenses in mouse lung [59]. In addition, the 8-OHdG levels, a parameter measured in the present contribution, provide an estimation of cancer risk. Recently, Wu and colleagues [60] detected a significant increase in the 8-OHdG content in leucocytes from cancer patients, as compared to healthy subjects. Evidence concerning a potential role of selenocyanates in the reduction of DNA oxidative modification, estimated as 8-OHdG levels, is scarce yet. Nevertheless, *p*-XSC was also showed decrease 8-OHdG levels in rat mammary glands, 6 hours after intragastric administration of 2-amino-1-methyl-6-phenylimidazo[4,5–*b*]pyridine—a well-known carcinogenic agent [61, 62]. Other organoselenium compound able to decrease 8-OHdG content in animals is (PhSe)_2_ (5 *μ*mol/kg body weight), as described in the brain of mice orally receiving methylmercury, which was accompanied by decreased brain-derived neurotrophic factor level, oxidative disturbances, and histological modifications in the cerebral cortex [63]. Selenocyanates can also reduce genotoxicity induced by certain compounds *in vivo*. To illustrate, oral administration of diphenylmethyl selenocyanate produced a significant decrease in the DNA damage in hepatocytes of mice acutely receiving carbon tetrachloride [64]. Thereby, Compound 3b, Compound 3d, Compound 3e, and Compound 3f have potential antigenotoxic activity by mitigating the increase in 8-OHdG content induced by H_2_O_2_ in the cell cultures.

## 5. Conclusions

In conclusion, we have described the synthesis and preclinical antioxidant assessment of novel selenocyanates (3a–f) using standardized and widely available methodologies. All selenocyanates were evaluated for their antioxidant ability in vitro. Compound 3b, Compound 3d, and Compound 3f showed significant activity when tested in cultured mouse neurons exposed to H_2_O_2_, with a pattern resembling to (PhSe)_2_. Measurement of CAT and SOD activities provided further evidence that most of the synthesized compounds are endowed of marked antioxidant activity. We did not use various concentrations of each compound in culture aiming to determine the best value with minimal potential adverse events (or a dose-response curve), which is one limitation of the present work. We argue that further in vitro studies using these promising selenocyanates will pave the way for their use in most sophisticated pathological experimental scenarios and potentially as a novel platform of antioxidant drugs with therapeutic properties.

## Figures and Tables

**Table 1:** Synthesis of organoselenium compounds (3a–f) from allylic bromides (2a–f). 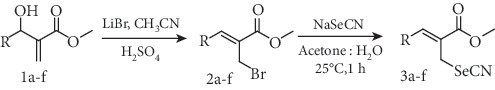

Entry	R	Yield (%)^[a]^ of 2	Yield (%)^[a]^ of 3
1	C_6_H_5_	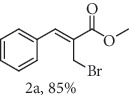	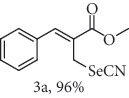

2	4-Br-C_6_H_4_	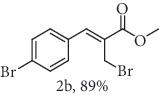	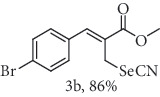

3	2-Br-C_6_H_4_	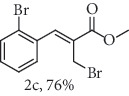	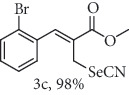

4	4-Cl-C_6_H_4_	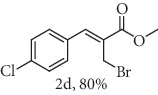	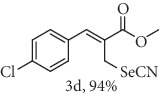

5	2,4-(Cl)_2_-C_6_H_3_	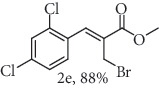	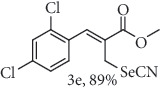

6	4-NO_2_-C_6_H_4_	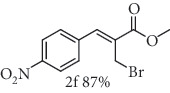	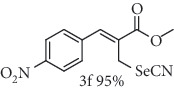

^[a]^Isolated yields.

## Data Availability

The data used to support the findings of this study are available from the corresponding authors upon request.

## Supplementary Materials

Supplementary Materials Figure S1: Nuclear Magnetic Resonance Spectra: (a) 1H NMR (200 MHz) in CDCl_3_ and (b) 13C NMR (50 MHz) in CDCl_3_ for compound 3a. Figure S2: Nuclear Magnetic Resonance Spectra: (a) 1H NMR (300 MHz) in CDCl_3_ and (b) 13C NMR (101 MHz) in CDCl_3_ for compound 3b. Figure S3: Nuclear Magnetic Resonance Spectra: (a) 1H NMR (400 MHz) in CDCl_3_ and (b) 13C NMR (101 MHz) in CDCl_3_ for compound 3c. Figure S4: Nuclear Magnetic Resonance Spectra: (a) 1H NMR (300 MHz) in CDCl_3_ and (b) 13C NMR (100 MHz) in CDCl_3_ for compound 3d. Figure S5: Nuclear Magnetic Resonance Spectra: (a) 1H NMR (400 MHz) in CDCl_3_ and (b) 13C NMR (101 MHz) in CDCl_3_ for compound 3e. Figure S6: Nuclear Magnetic Resonance Spectra: (a) 1H NMR (400 MHz) in CDCl3 and (b) 13C NMR (101 MHz) in CDCl_3_ for compound 3f. Figure S7: infrared spectra of compound 3a. Figure S8: infrared spectra of compound 3b. Figure S9: infrared spectra of compound 3c. Figure S10: infrared spectra of compound 3d. Figure S11: infrared spectra of compound 3e. Figure S12: infrared spectra of compound 3f. Figure S13: infrared spectra of starting material 2a. Figure S14: infrared spectra of starting material 2b. Figure S15: infrared spectra of starting material 2c. Figure S16: infrared spectra of starting material 2d. Figure S17: infrared spectra of starting material 2e. Figure S18: infrared spectra of starting material 2f.
