# Supplementary material for: Synthesis of Novel Selenocyanates and Evaluation of Their Effect in Cultured Mouse Neurons Submitted to Oxidative Stress
Source: Oxid Med Cell Longev. 2020 May 28;2020:5417024. doi: 10.1155/2020/5417024 (PMC7275203; doi:10.1155/2020/5417024)

SUPPORTING INFORMATION

Synthesis of novel selenocyanates and evaluation of their effect in cultured mouse neurons submitted to oxidative stress

Tiago E. A. Frizon,*[,1] José H. Cararo,[2] Sumbal Saba,*[,3] Gustavo C. Dal-Pont,[2] Monique Michels,[4] Hugo de C. Braga,[5] Tairine Pimentel,[6] Felipe Dal-Pizzol,[4] Samira S. Valvassori*[,2] and Jamal Rafique*[,6]

[1] Department of Energy and Sustainability, Federal University of Santa Catarina (UFSC), Araranguá, 88906-072, SC, Brazil.
[2] Translational Psychiatry Laboratory, Graduate Program in Health Sciences, University of Southern Santa Catarina (UNESC), Criciúma, 88806-000, SC, Brazil.
[3] Center for Natural and Human Sciences-CCNH, Federal University of ABC (UFABC), Santo André, 09210-580, SP, Brazil.
[4] Laboratory of Experimental Pathophysiology, Graduate Program in Health Sciences, Health Sciences Unit, University of Southern Santa Catarina (UNESC), Criciúma. 88806-000 SC, Brazil.
[5] Federal University of São Paulo (UNIFESP), São José dos Campos, 12231-280 SP, Brazil.
[6] Institute of Chemistry, Federal University of Mato Grosso do Sul (UFMS), Campo Grande, 79074-460, MS, Brazil.

Tiago E. A. Frizon: tiago.frizon@ufsc.br ;
Sumbal Saba: sumbal6s@gmail.com ; sumbal.saba@ufabc.edu.br
Samira S. Valvassori: samiravalvassori@unesc.net ;
* Jamal Rafique: jamal.chm@gmail.com, jamal.rafique@ufms.br

TABLE OF CONTENTS

I. NMR spectra of the products **3a-f** 2

II. FTIR spectra of the products **3a-f** 8

III. FTIR spectra of the starting materials **2a-f** 11

(I) NMR spectra of the products 3a-f:

Figure S1- Nuclear Magnetic Resonance Spectra: **(a)** ^{1}H NMR (200 MHz) in $CDCl_3$ and **(b)** ^{13}C NMR (50 MHz) in $CDCl_3$ for compound **3a**.

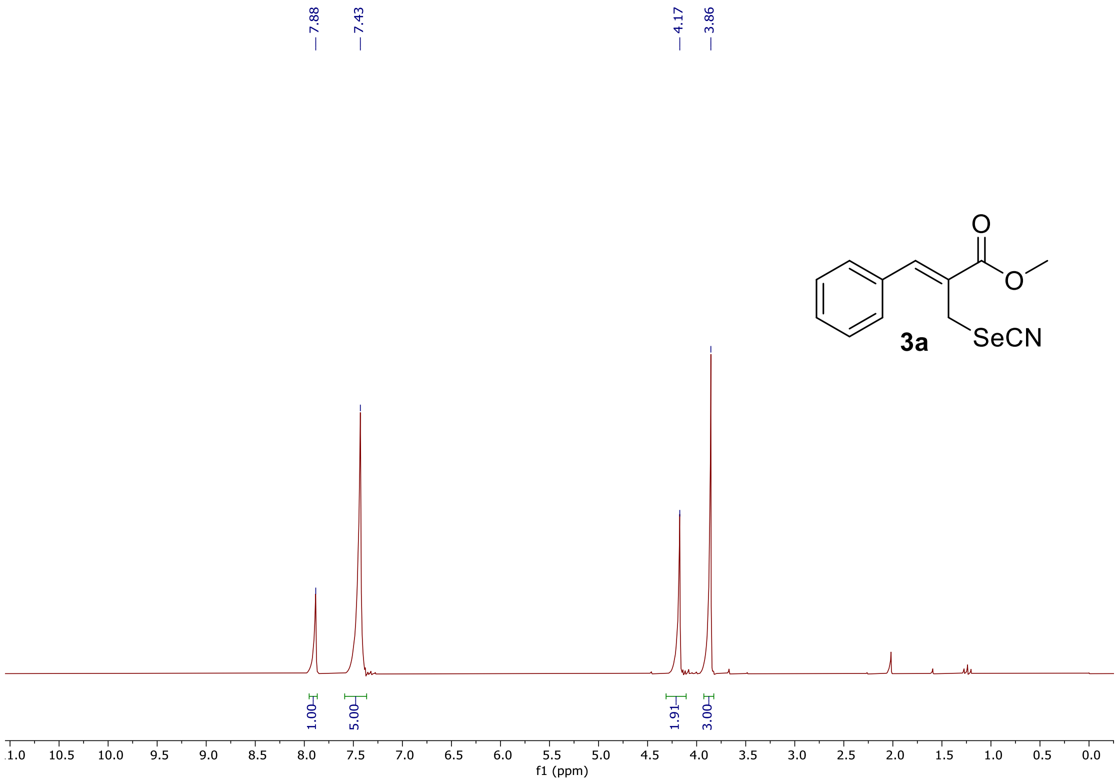

(a)

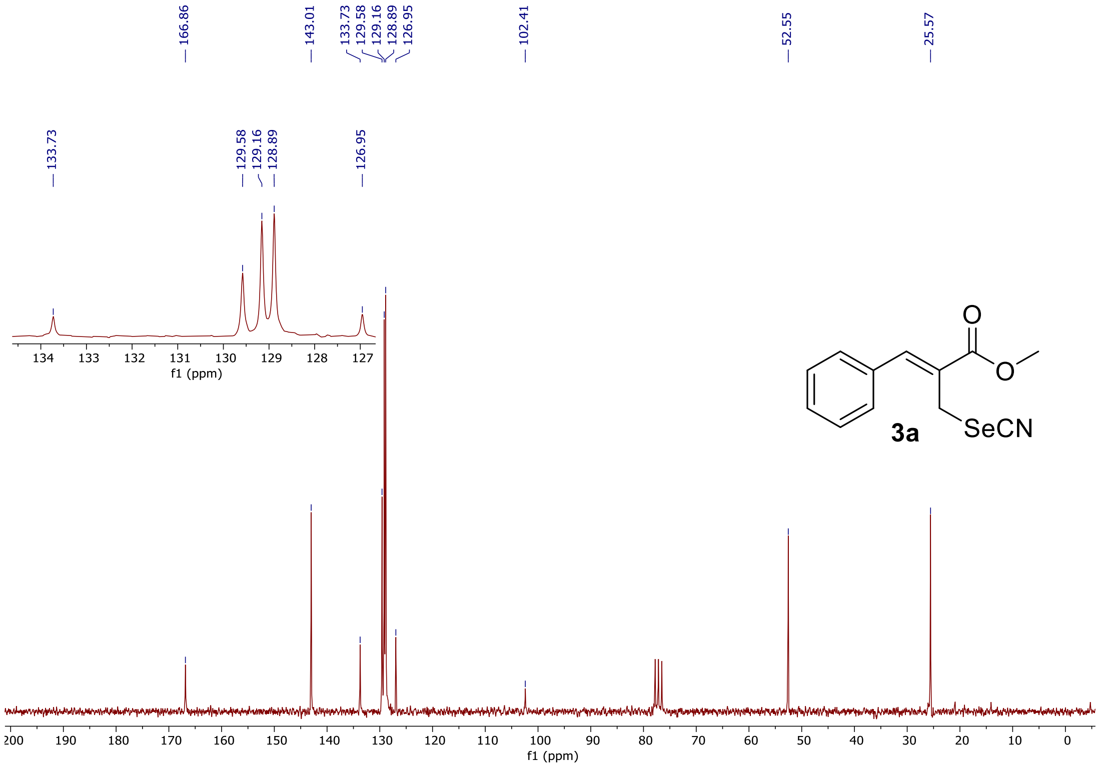

(b)

Figure S2- Nuclear Magnetic Resonance Spectra: **(a)** ^{1}H NMR (300 MHz) in $CDCl_3$ and **(b)** ^{13}C NMR (101 MHz) in $CDCl_3$ for compound **3b**.

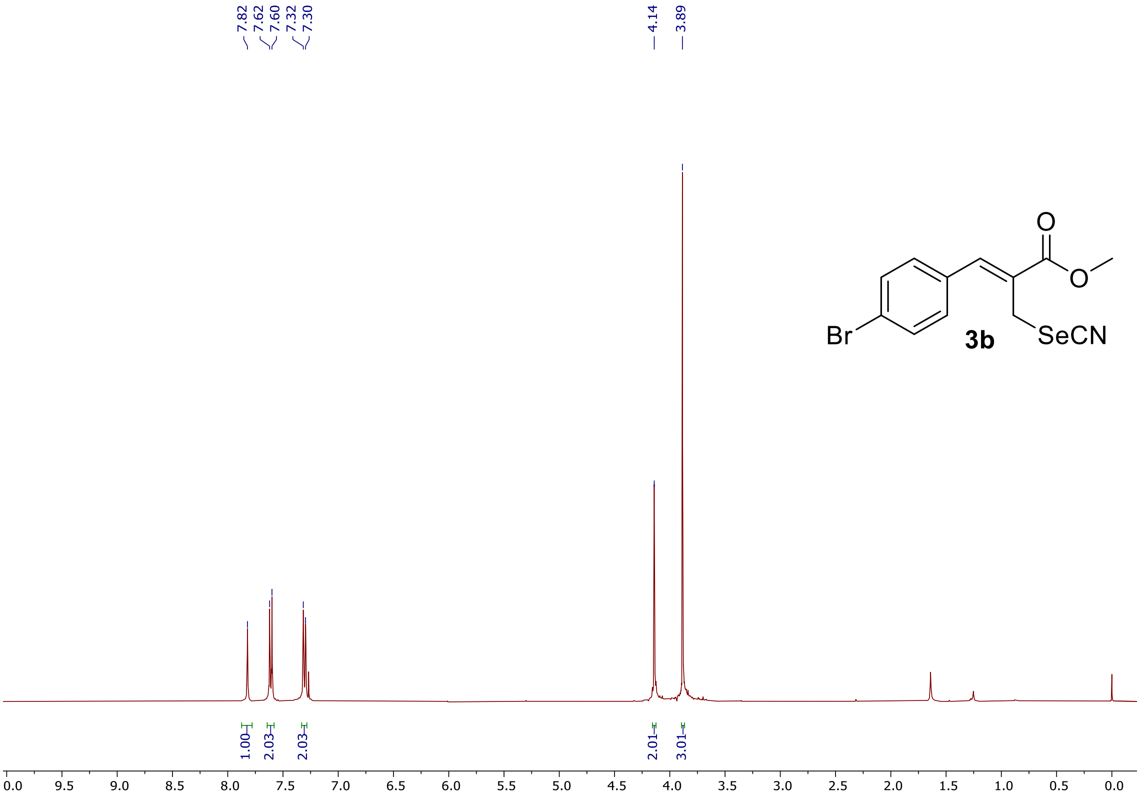

(a)

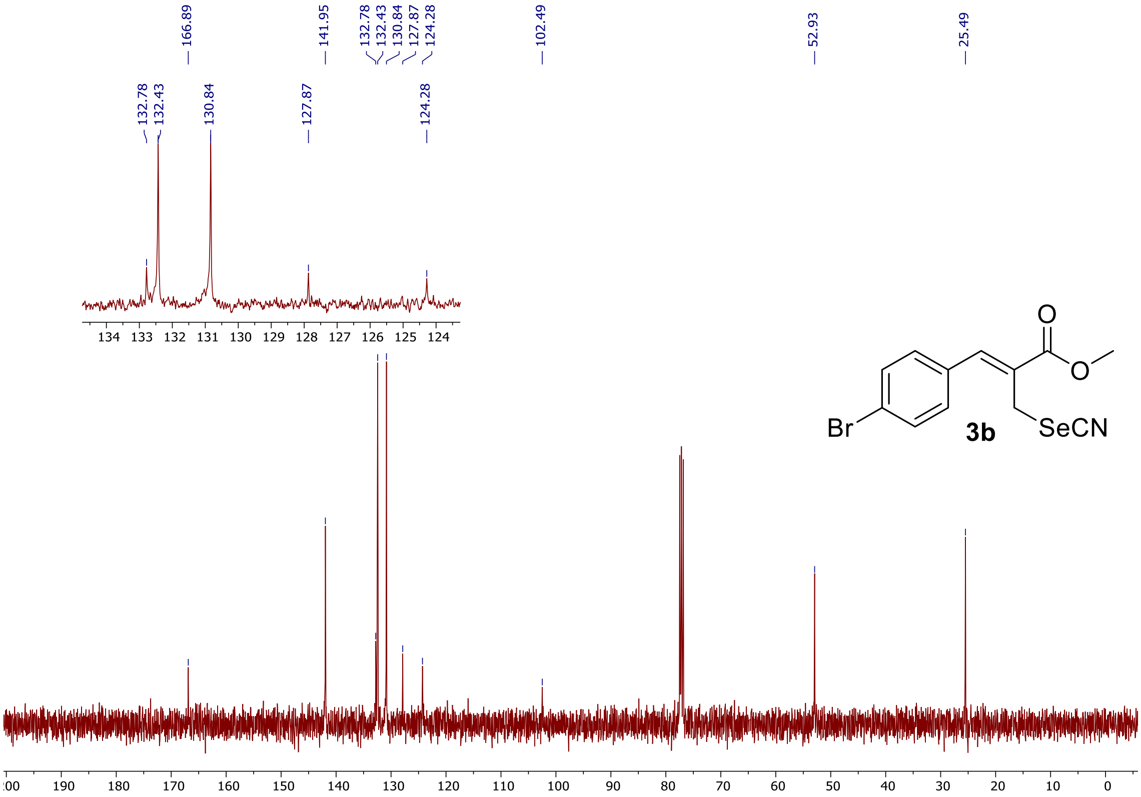

(b)

Figure S3- Nuclear Magnetic Resonance Spectra: **(a)** ^{1}H NMR (400 MHz) in $CDCl_3$ and **(b)** ^{13}C NMR (101 MHz) in $CDCl_3$ for compound **3c**.

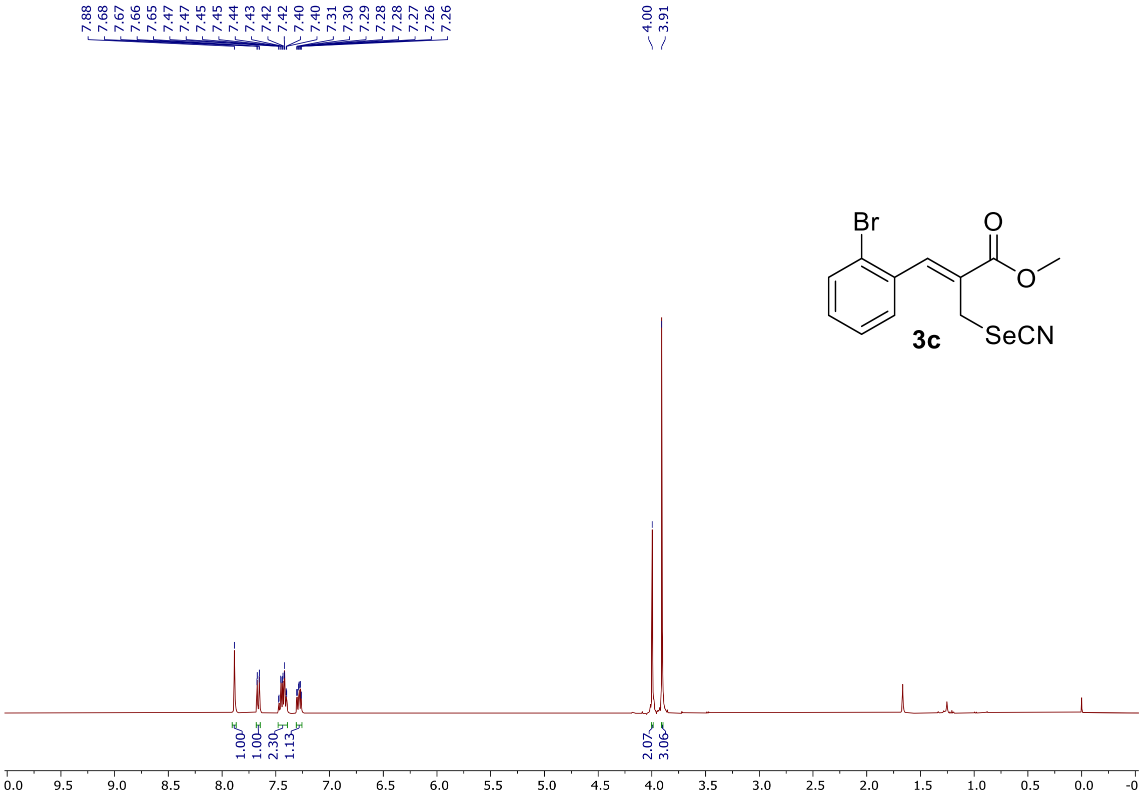

(a)

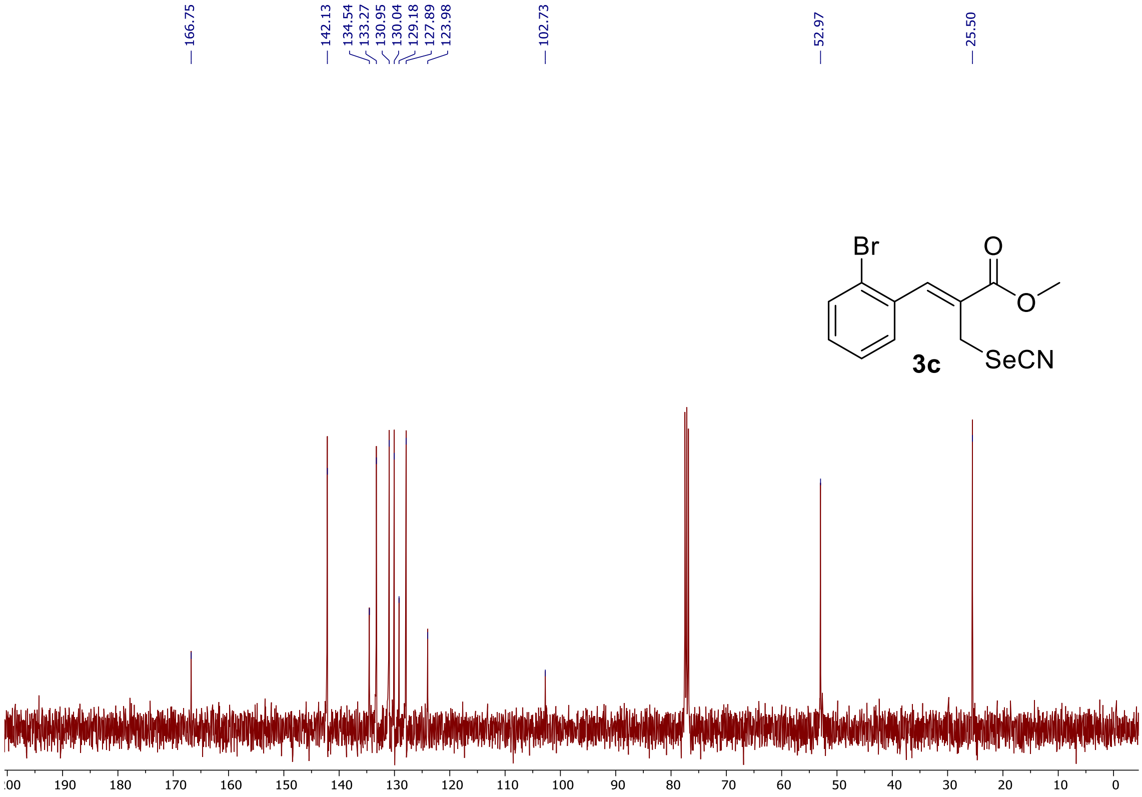

(b)

Figure S4- Nuclear Magnetic Resonance Spectra: **(a)** ^{1}H NMR (300 MHz) in $CDCl_3$ and **(b)** ^{13}C NMR (100 MHz) in $CDCl_3$ for compound **3d**.

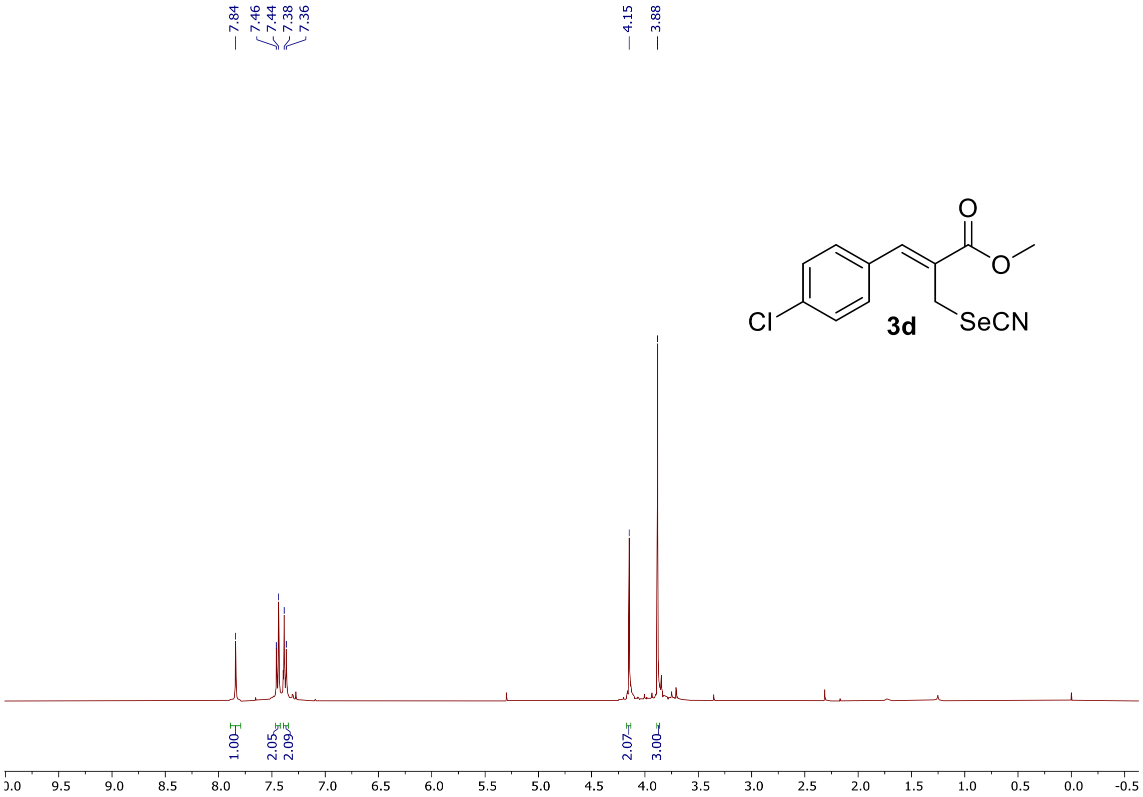

(a)

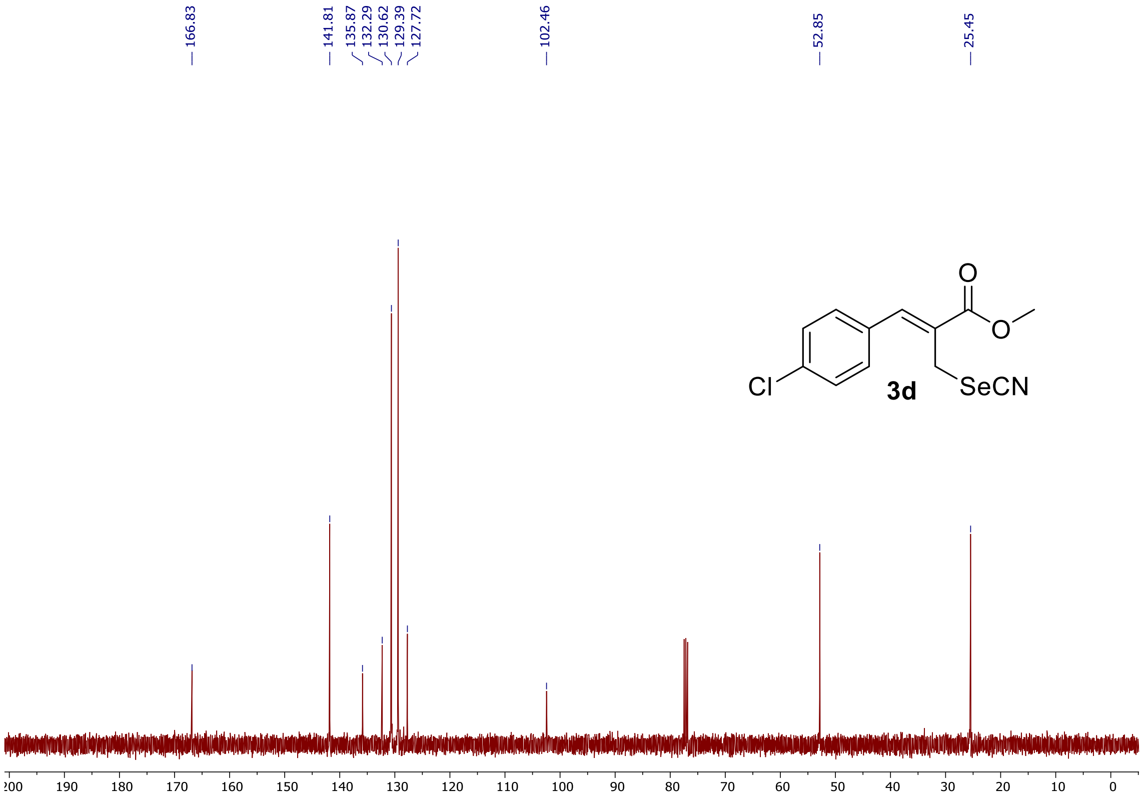

(b)

Figure S5- Nuclear Magnetic Resonance Spectra: **(a)** ^{1}H NMR (400 MHz) in $CDCl_3$ and **(b)** ^{13}C NMR (101 MHz) in $CDCl_3$ for compound **3e**.

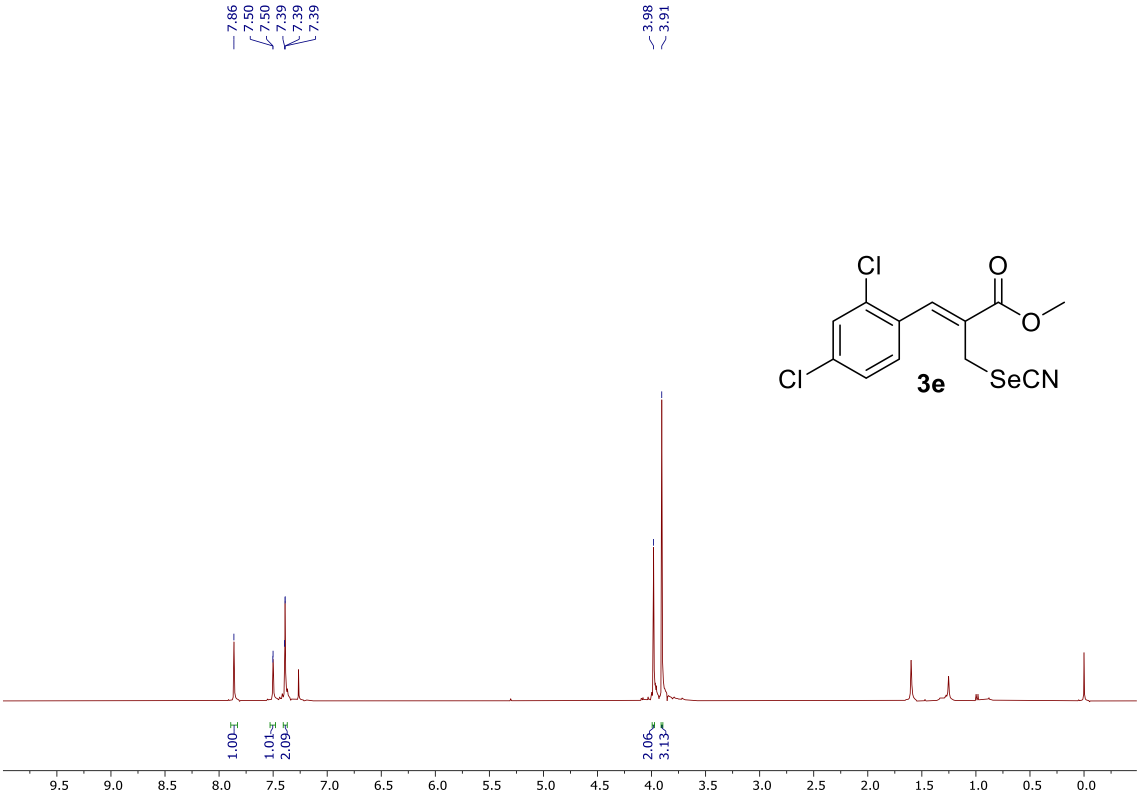

(a)

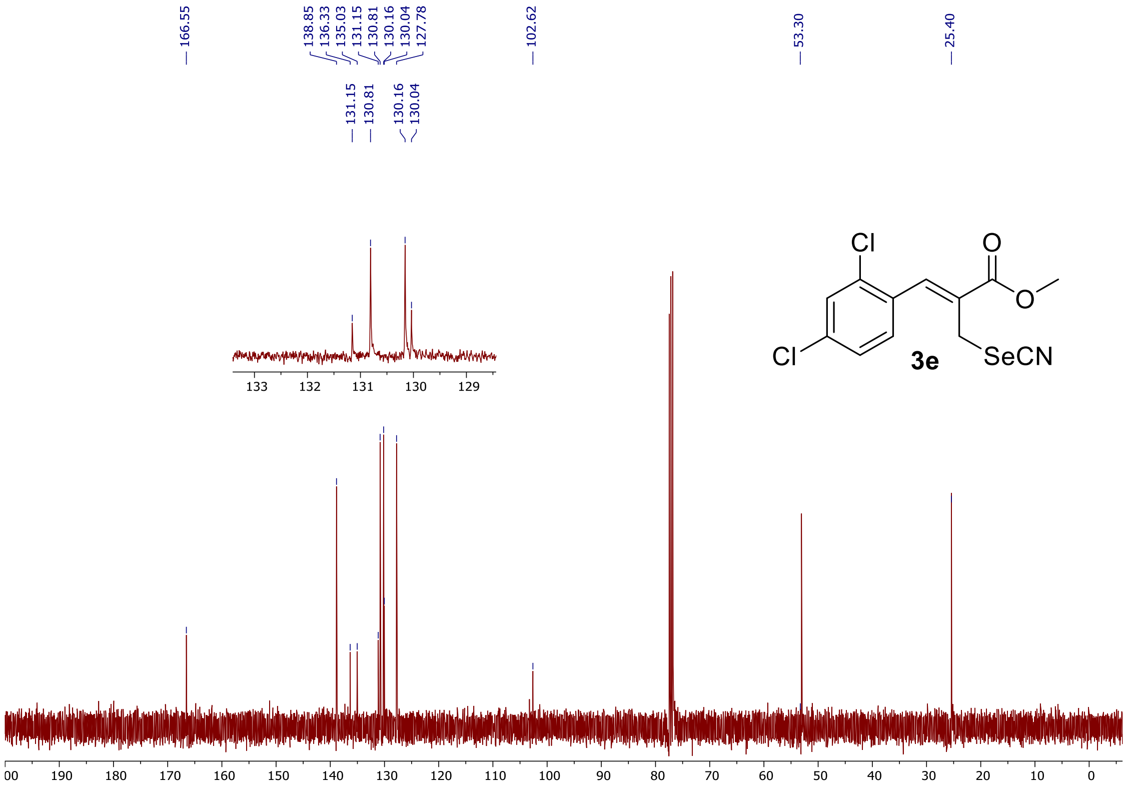

(b)

Figure S6- Nuclear Magnetic Resonance Spectra: **(a)** ^{1}H NMR (400 MHz) in $CDCl_3$ and **(b)** ^{13}C NMR (101 MHz) in $CDCl_3$ for compound **3f**.

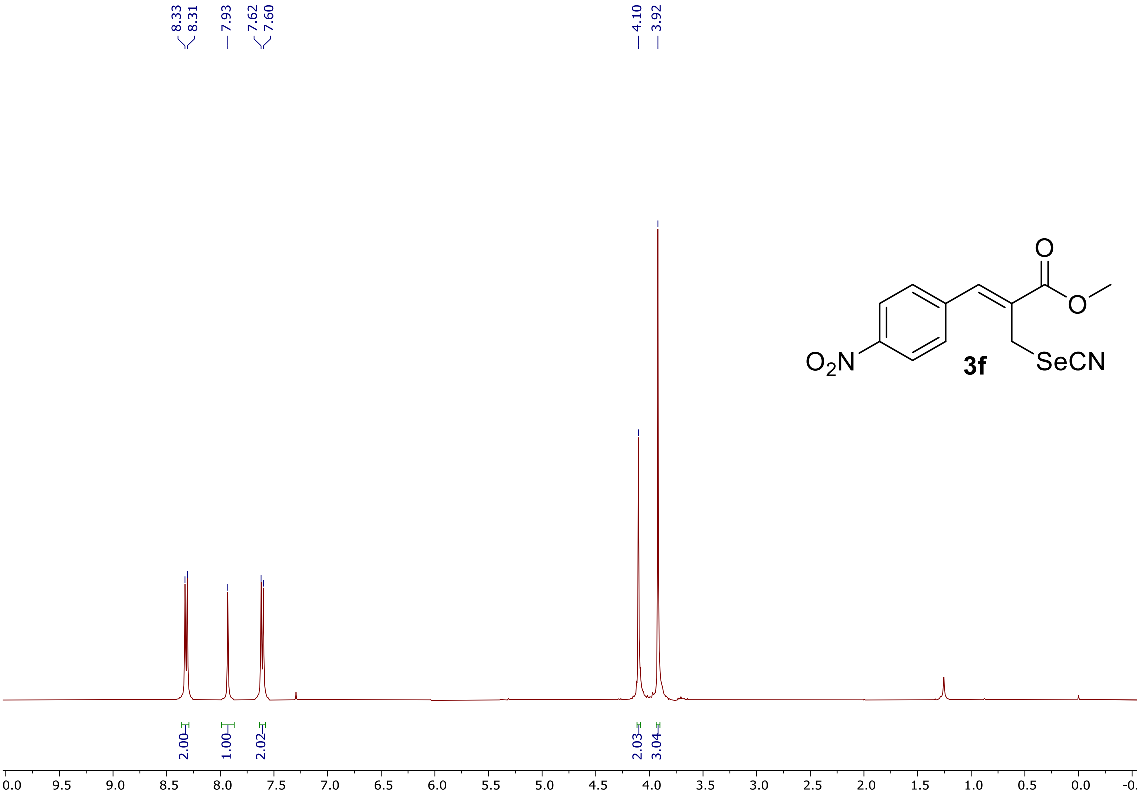

(a)

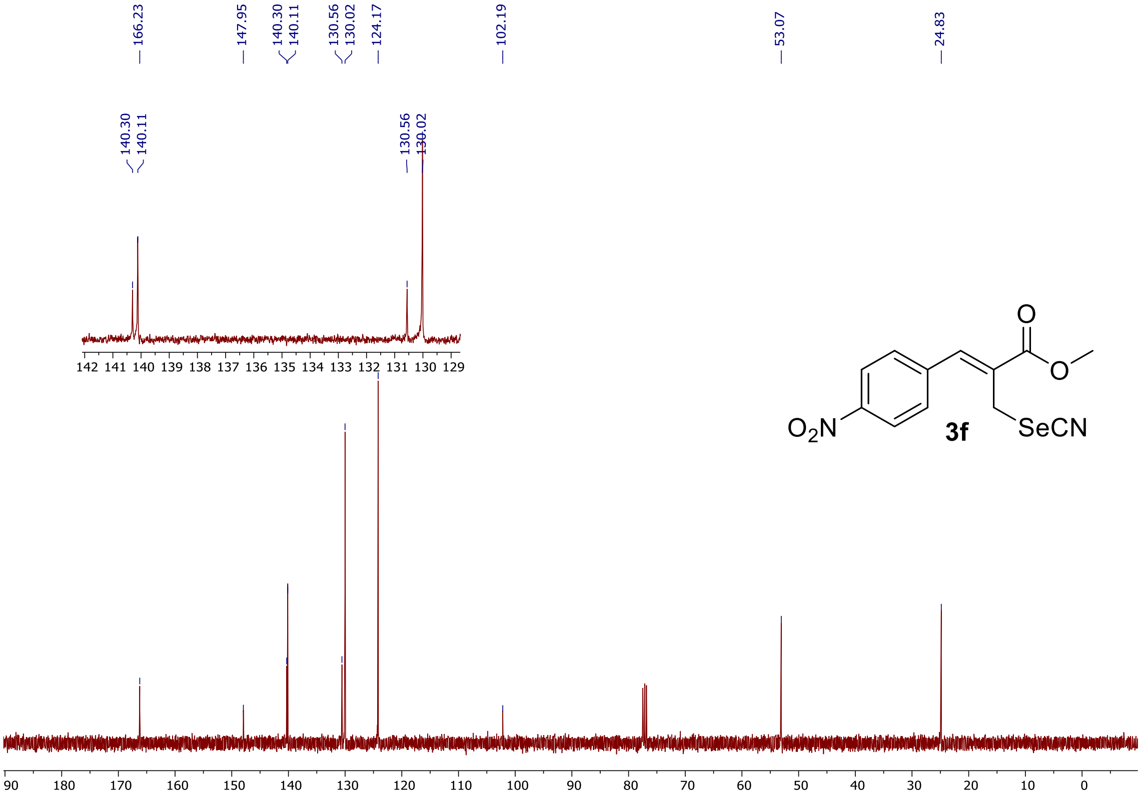

(b)

(II) FTIR spectra of the products 3a-f:

Figure S7- Infrared spectra of compound **3a.**

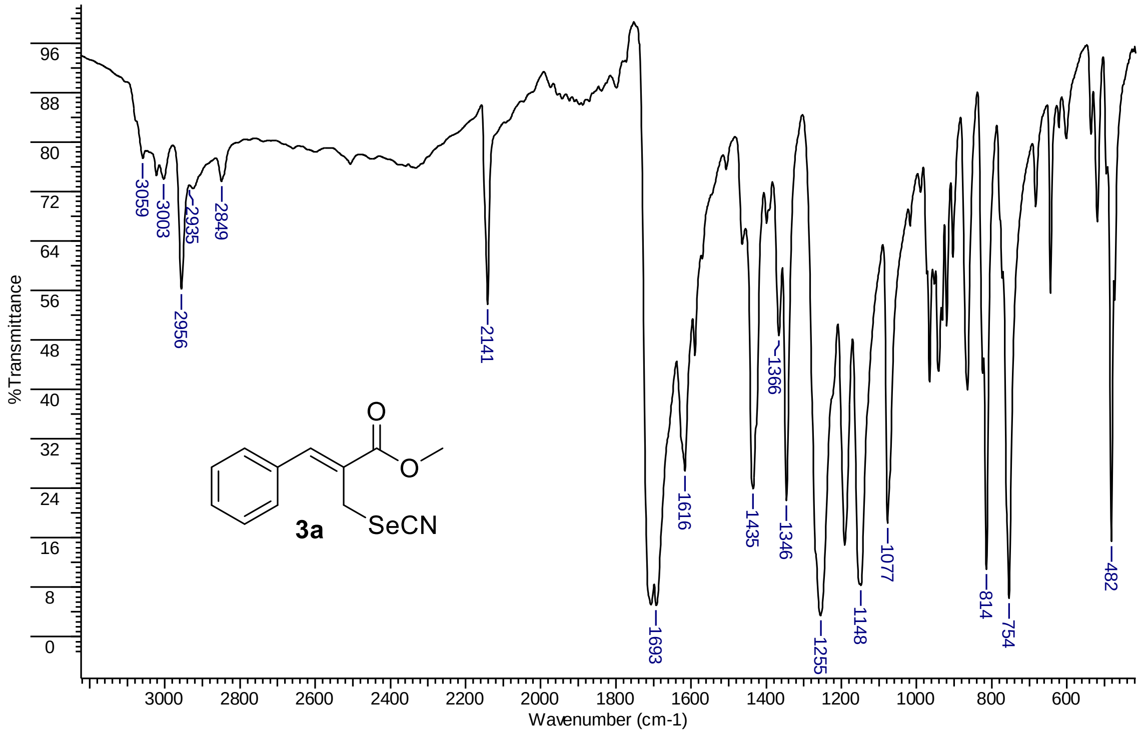

Figure S8- Infrared spectra of compound **3b.**

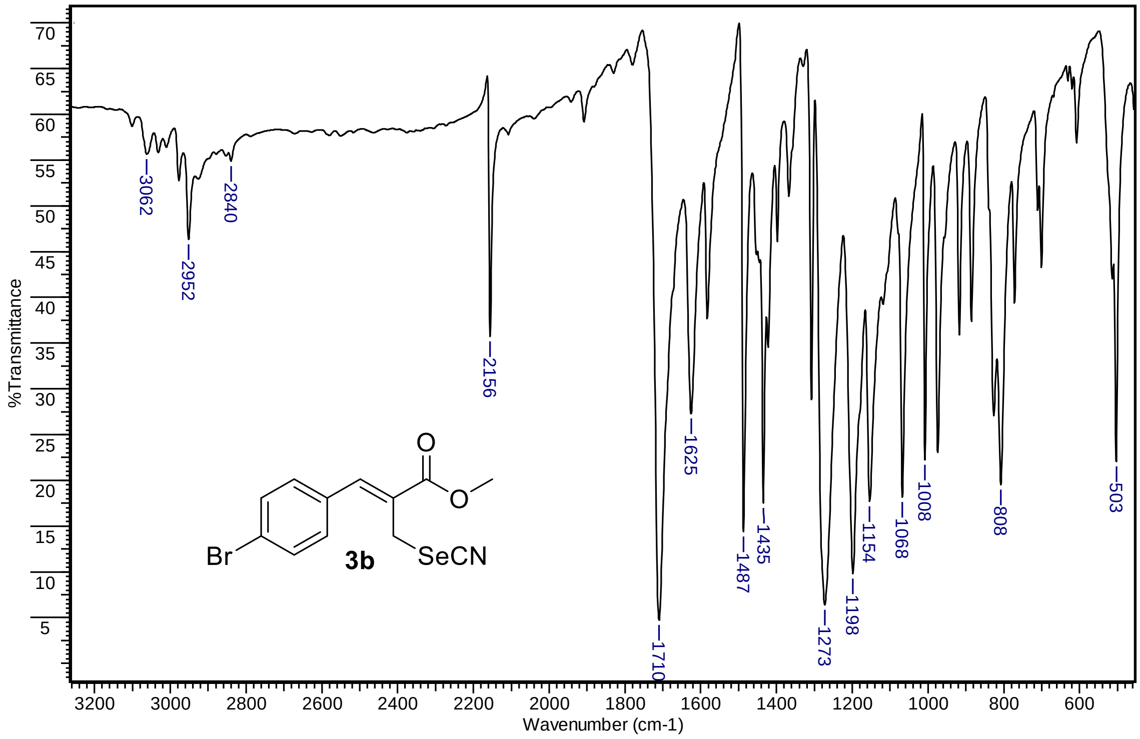

Figure S9- Infrared spectra of compound **3c.**

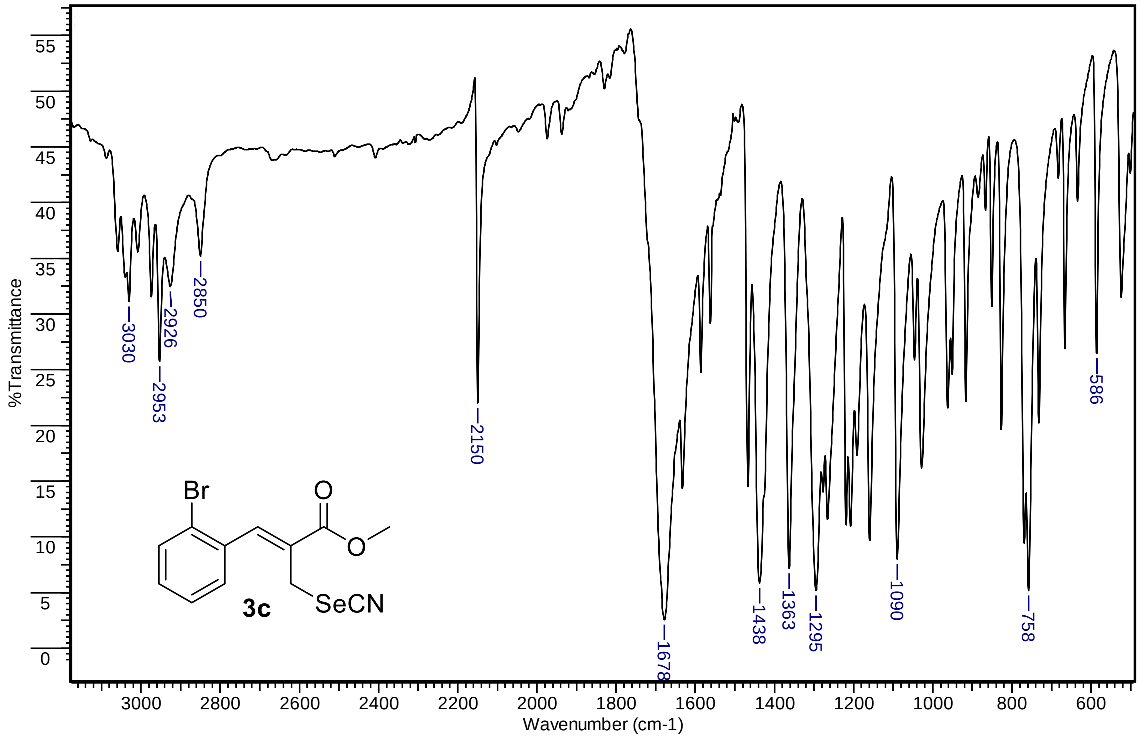

Figure S10- Infrared spectra of compound **3d.**

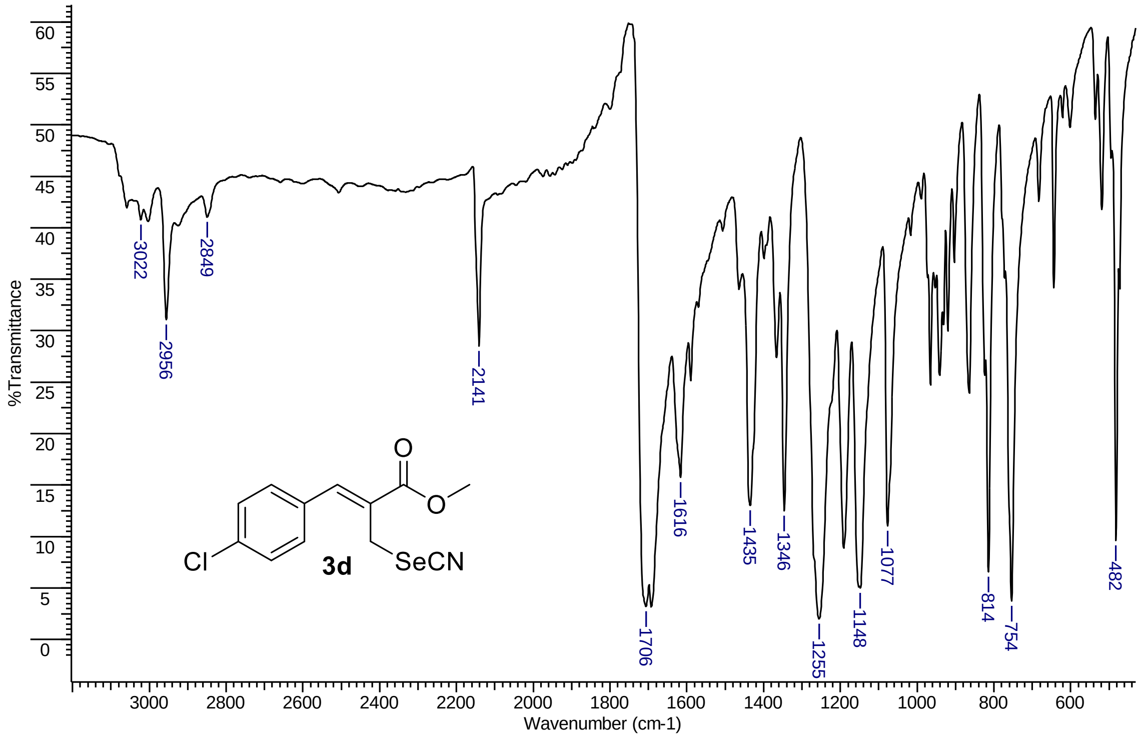

Figure S11- Infrared spectra of compound **3e.**

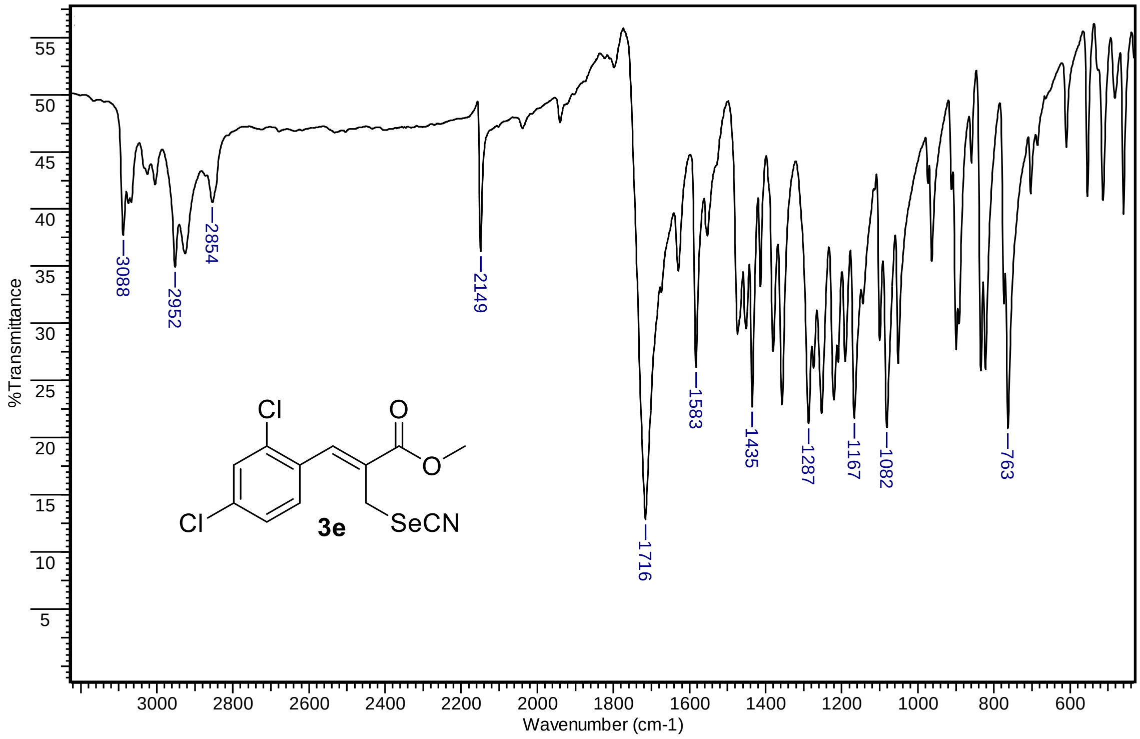

Figure S12- Infrared spectra of compound **3f.**

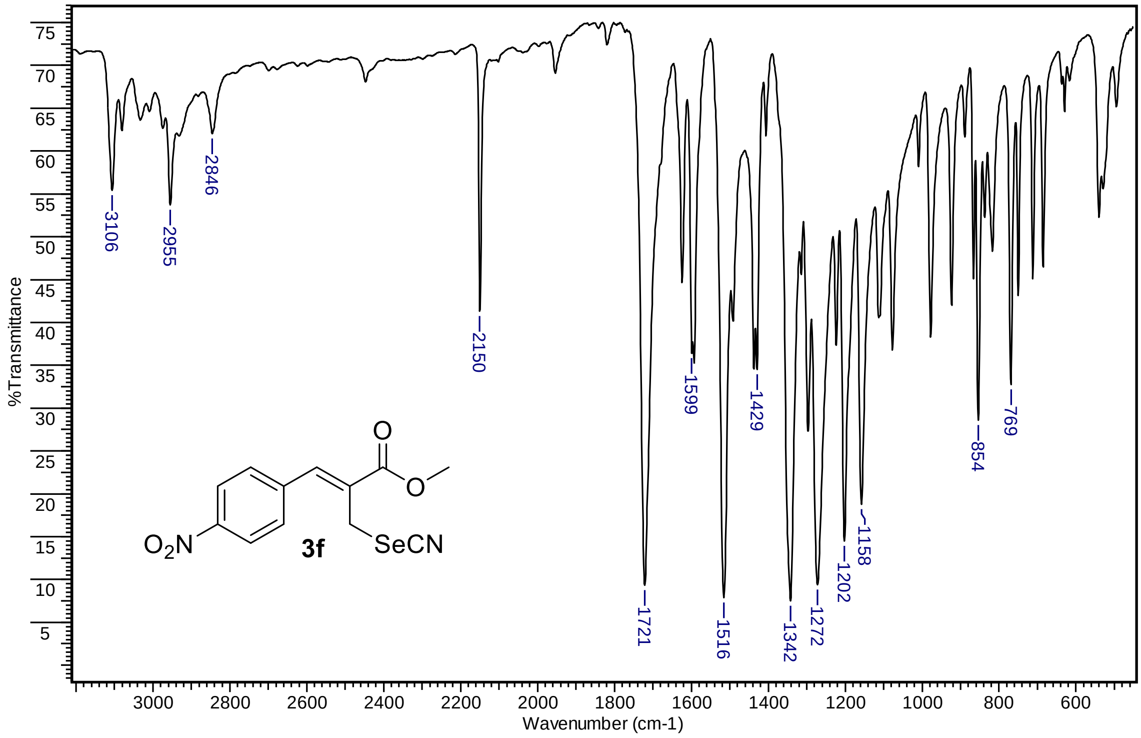

(III) FTIR spectra of the starting materials 2a-f:

Figure S13- Infrared spectra of starting material **2a.**

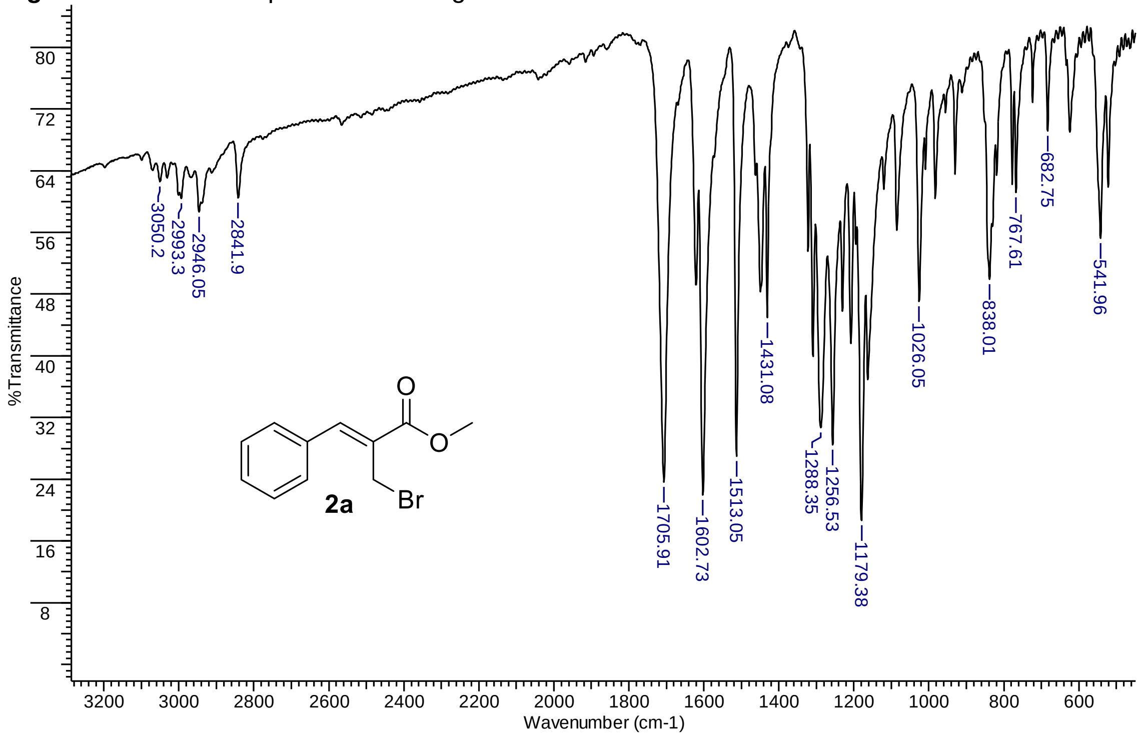

Figure S14- Infrared spectra of starting material **2b.**

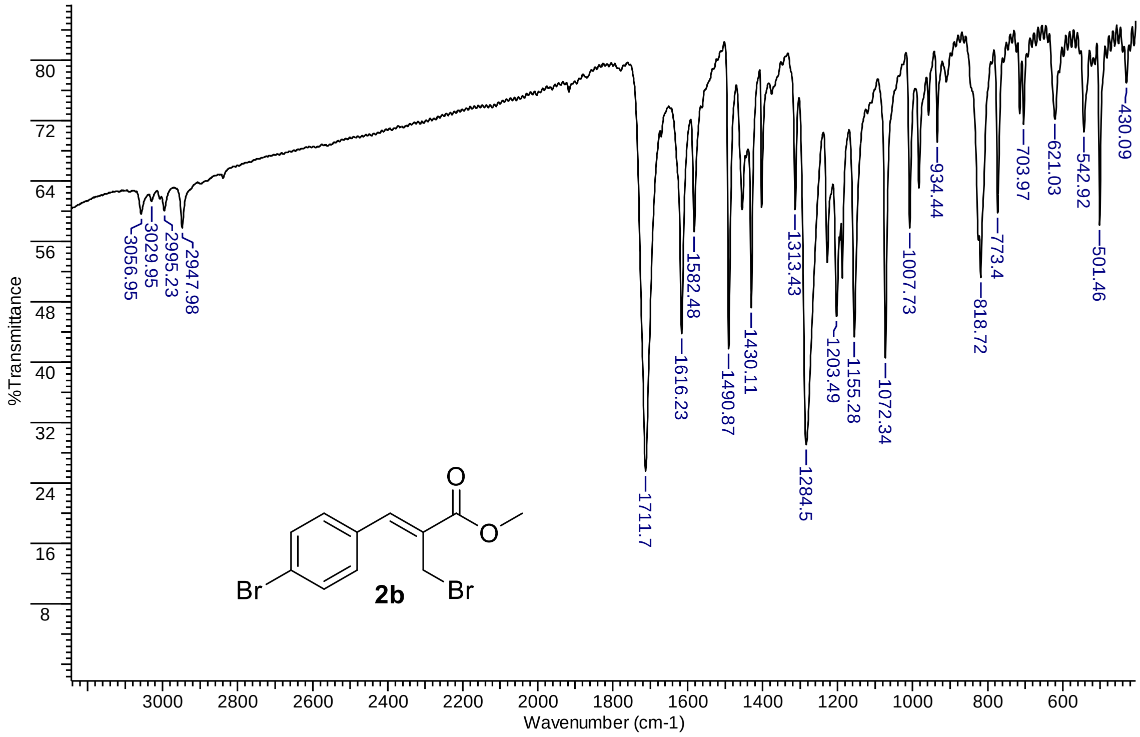

Figure S15- Infrared spectra of starting material **2c.**

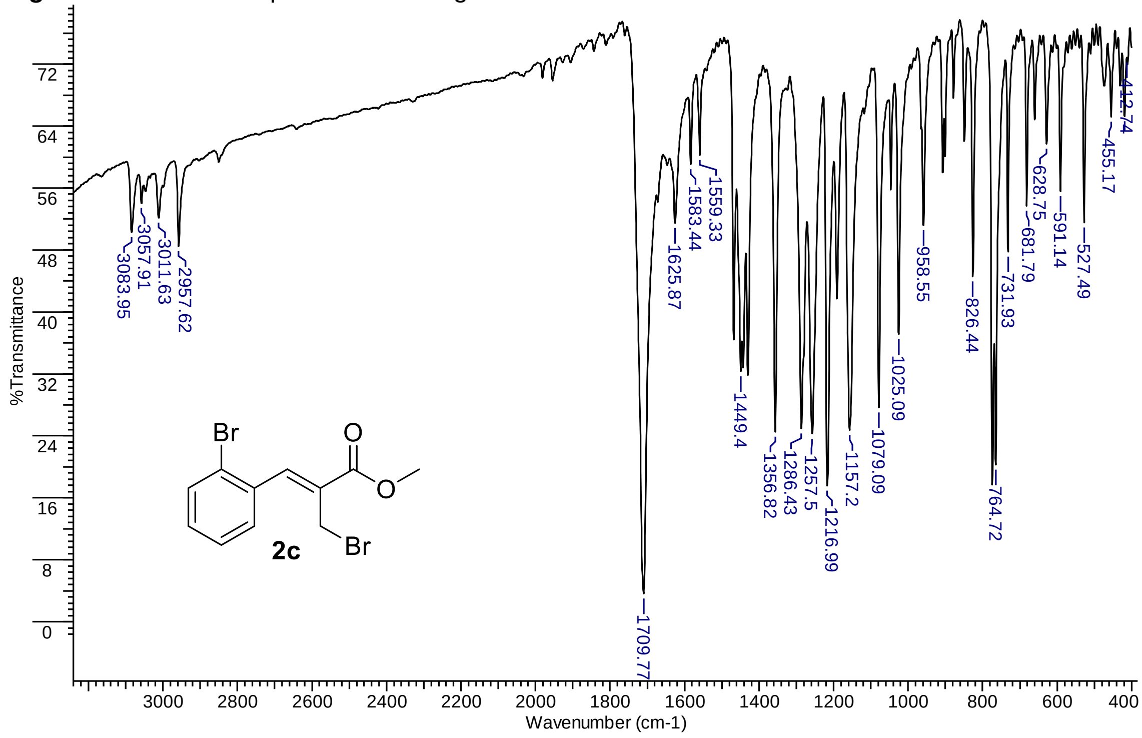

Figure S16- Infrared spectra of starting material **2d.**

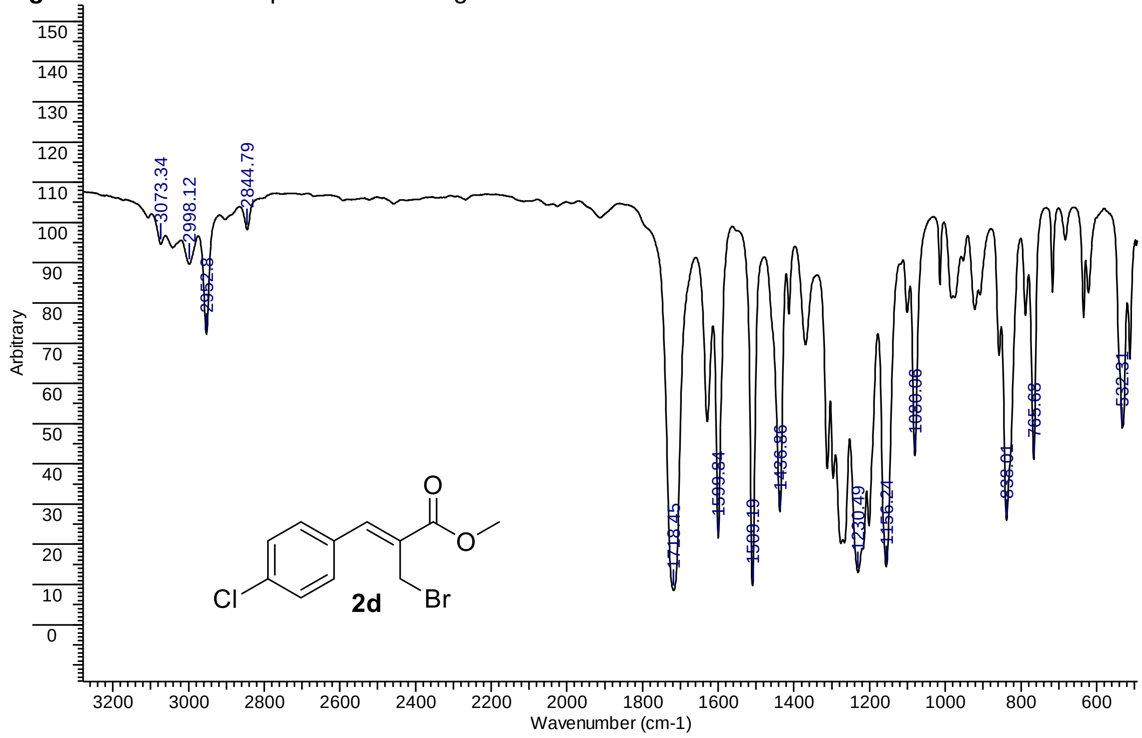

Figure S17- Infrared spectra of starting material **2e.**

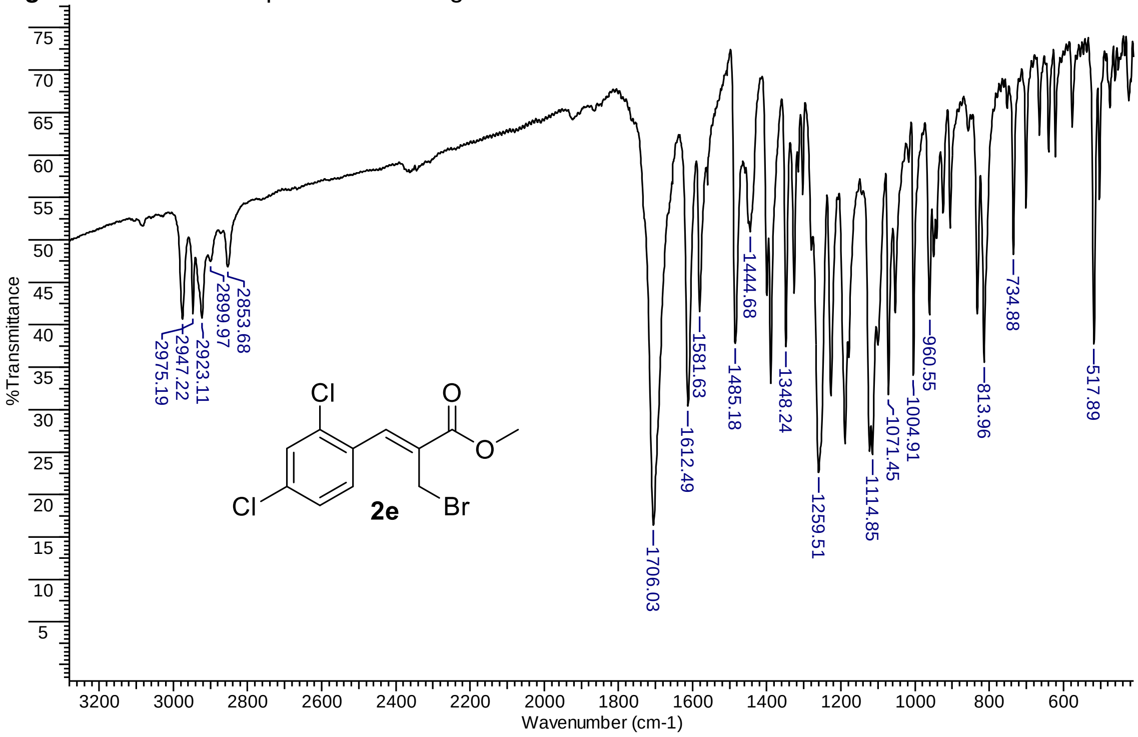

Figure S18- Infrared spectra of starting material **2f.**

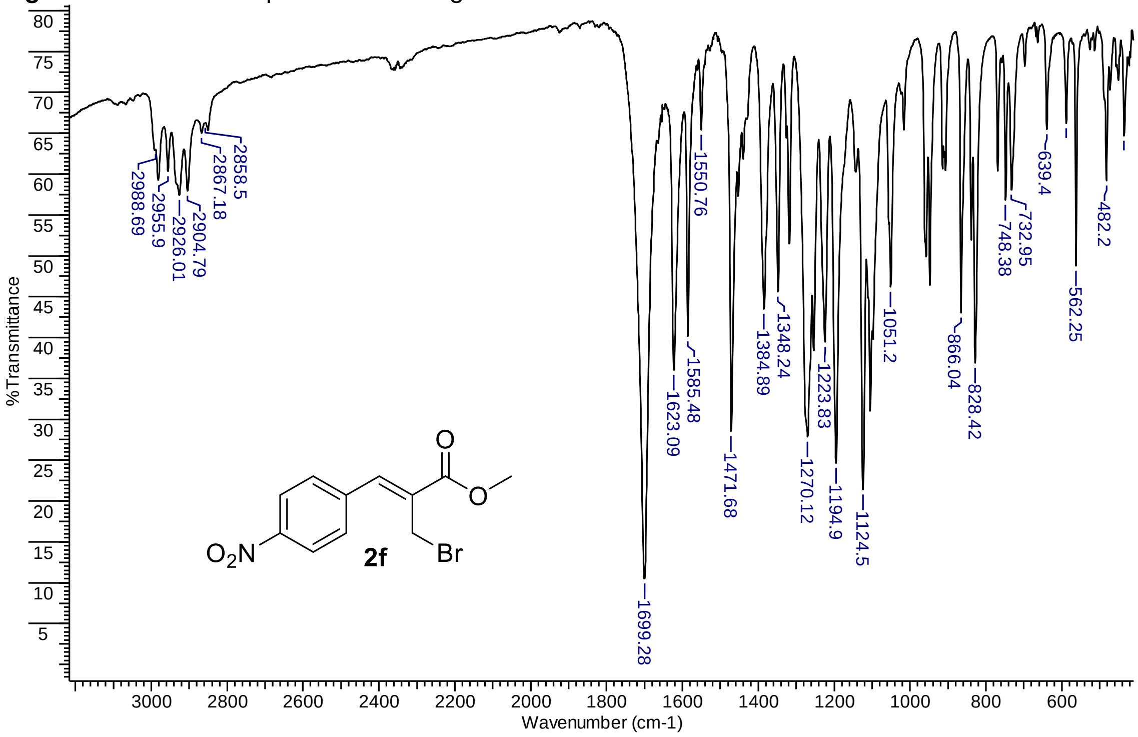

Supplement: Supplementary Materials — Figure S1: Nuclear Magnetic Resonance Spectra: (a) 1H NMR (200 MHz) in CDCl3 and (b) 13C NMR (50 MHz) in CDCl3 for compound 3a. Figure S2: Nuclear Magnetic Resonance Spectra: (a) 1H NMR (300 MHz) in CDCl3 and (b) 13C NMR (101 MHz) in CDCl3 for compound 3b. Figure S3: Nuclear Magnetic Resonance Spectra: (a) 1H NMR (400 MHz) in CDCl3 and (b) 13C NMR (101 MHz) in CDCl3 for compound 3c. Figure S4: Nuclear Magnetic Resonance Spectra: (a) 1H NMR (300 MHz) in CDCl3 and (b) 13C NMR (100 MHz) in CDCl3 for compound 3d. Figure S5: Nuclear Magnetic Resonance Spectra: (a) 1H NMR (400 MHz) in CDCl3 and (b) 13C NMR (101 MHz) in CDCl3 for compound 3e. Figure S6: Nuclear Magnetic Resonance Spectra: (a) 1H NMR (400 MHz) in CDCl3 and (b) 13C NMR (101 MHz) in CDCl3 for compound 3f. Figure S7: infrared spectra of compound 3a. Figure S8: infrared spectra of compound 3b. Figure S9: infrared spectra of compound 3c. Figure S10: infrared spectra of compound 3d. Figure S11: infrared spectra of compound 3e. Figure S12: infrared spectra of compound 3f. Figure S13: infrared spectra of starting material 2a. Figure S14: infrared spectra of starting material 2b. Figure S15: infrared spectra of starting material 2c. Figure S16: infrared spectra of starting material 2d. Figure S17: infrared spectra of starting material 2e. Figure S18: infrared spectra of starting material 2f. [file 5417024.f1.pdf]
